# Unraveling substituent effects on the glass transition temperatures of biorenewable polyesters

**DOI:** 10.1038/s41467-018-05269-3

**Published:** 2018-07-23

**Authors:** Xiaopeng Yu, Junteng Jia, Shu Xu, Ka Un Lao, Maria J. Sanford, Ramesh K. Ramakrishnan, Sergei I. Nazarenko, Thomas R. Hoye, Geoffrey W. Coates, Robert A. DiStasio

**Affiliations:** 1 Department of Chemistry and Chemical Biology, Cornell University, Ithaca, NY 14853 USA; 2 Department of Chemistry, University of Minnesota, Minneapolis, MN 55455 USA; 3 School of Polymers and High Performance Materials, University of Southern Mississippi, Hattiesburg, MS 39402 USA

## Abstract

Converting biomass-based feedstocks into polymers not only reduces our reliance on fossil fuels, but also furnishes multiple opportunities to design biorenewable polymers with targeted properties and functionalities. Here we report a series of high glass transition temperature (*T*_g_ up to 184 °C) polyesters derived from sugar-based furan derivatives as well as a joint experimental and theoretical study of substituent effects on their thermal properties. Surprisingly, we find that polymers with moderate steric hindrance exhibit the highest *T*_g_ values. Through a detailed Ramachandran-type analysis of the rotational flexibility of the polymer backbone, we find that additional steric hindrance does not necessarily increase chain stiffness in these polyesters. We attribute this interesting structure-property relationship to a complex interplay between methyl-induced steric strain and the concerted rotations along the polymer backbone. We believe that our findings provide key insight into the relationship between structure and thermal properties across a range of synthetic polymers.

## Abstract (web summary)

Biorenewable feedstocks allow the design of polymers with targeted properties and functionalities. Here the authors report a series of high glass transition temperature sugar-based polyesters and investigate how substituents affect their thermal properties.

## Introduction

As the drive for sustainability continues, petroleum-derived plastics are steadily being replaced by alternative bio-based polymers^1,2^. In this regard, the conversion of biorenewable feedstocks into aliphatic polyesters has become an especially desirable path forward as aliphatic polyesters are in general both biodegradable and biocompatible^3^. During the past decade, there has been growing interest in developing synthetic strategies for copolymerizing epoxides and cyclic anhydrides to make such aliphatic polyesters^4,5^. In addition to providing numerous opportunities for incorporating biorenewable content into polymers, these distinct monomer sets also allow us to tune a myriad of polymer properties and functionalities for different applications. For example, we recently found that bulky terpene-based anhydrides can be employed to make aliphatic polyesters with glass transition temperatures (*T*_g_) up to 184 °C, thereby positioning them for use in a number of high-temperature applications^6,7^. Inspired by this finding, we decided to explore even more abundant biorenewable feedstocks as building blocks for high-*T*_g_ materials.

Polymers with high *T*_g_ values often have rigid backbones, which inhibit rotational flexibility in these polymer chains^8,9^. Hence, one of the most effective ways to increase chain rigidity is by the introduction of non-flexible ring structures into the polymer backbone, thereby hindering (or even eliminating in some cases) rotational flexibility^8^. As such, aromatic and aliphatic rings are often found as components of high-*T*_g_ polymers such as aromatic polycarbonates, polynorbornenes, and polyimides^8^. Another common way to increase *T*_g_ is by introducing substituents along/near the polymer backbone to hinder main-chain rotations through steric interactions^8,10^. Notable examples of such polymers include poly(α-methylstyrene), poly(2-methylstyrene), and poly(2,6-dimethylstyrene), all of which are characterized by *T*_g_ values that are significantly higher than their less substituted analogs^11^.

Following these design principles, we report herein several biorenewable polyesters with ring structures derived from furan, 2-methylfuran, and 2,5-dimethylfuran, all of which can be readily synthesized from pentoses and hexoses^12–14^. In doing so, we observe a quite unexpected structure-property relationship in this class of polymers, namely that the introduction of methyl substituents along the polymer backbone does not always increase *T*_g_ and an intermediate number of methyl substituents actually yields polymers with the highest *T*_g_ values. By performing a detailed theoretical analysis of the conformational flexibility in these polyesters, we provide key chemical and physical insight into the critical role played by methyl-induced steric strain in governing concerted rotations along the polymer chain. In particular, we demonstrate that methyl-induced strain can be leveraged to control chain flexibility in this class of polyesters by selectively destabilizing the relevant minimum-energy conformations across the rotational potential energy landscape. On the basis of these findings, we propose several simple and intuitive design principles for how methyl substitution can be used to rationalize and tune *T*_g_ trends across a range of synthetic polymers.

## Results

### Monomer synthesis

**Fig. 1:**
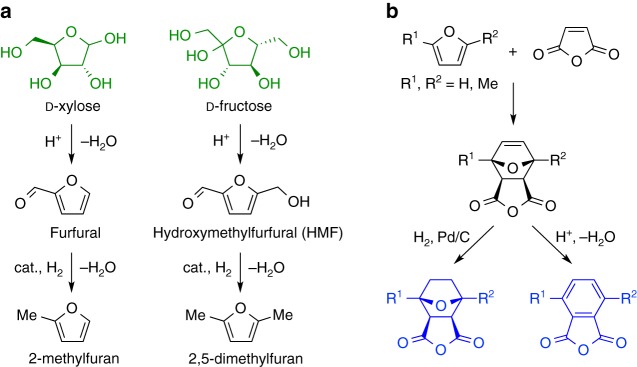
Monomer synthesis. **a** Catalytic conversion of d-xylose and d-fructose into 2-methylfuran and 2,5-dimethylfuran, respectively. **b** Transformation of substituted furan derivatives into tricyclic (left) and phthalic (right) anhydride monomers

Acid-catalyzed dehydration of pentoses (such as xylose) to furfural, and hexoses (such as glucose and fructose) to hydroxymethylfurfural (HMF), represent promising pathways for the utilization of carbohydrates (Fig. 1a)^12–14^. Although many biomass transformations require fermentation, this dehydration protocol proceeds without the use of enzymes, allowing it to be competitive in both cost and productivity^13,14^. As a result, the transformation of furfural and HMF into other value-added products and commodities has been the subject of extensive study^13,14^. For example, the hydrogenation of furfural and HMF yields 2-methylfuran and 2,5-dimethylfuran, respectively, both of which are regarded as potential biofuels (Fig. 1a)^12–14^.

In order to incorporate these sugar-derived biorenewable feedstocks into polymers, we investigated the Diels-Alder (D-A) reactions of a number of furan derivatives with maleic anhydride. As previously observed with furfural and maleimide, both furfural and HMF also do not readily undergo a D-A reaction with maleic anhydride, and we attribute this low reactivity to the presence of the electron-withdrawing aldehyde group and associated higher resonance stabilization in these compounds^15^. In contrast, 2-methylfuran and 2,5-dimethylfuran readily react with maleic anhydride to yield thermally labile unsaturated D-A adducts, which can then be hydrogenated or dehydrated to make tricyclic or phthalic anhydrides, respectively, with a targeted number of methyl substituents (Fig. 1b)^16–18^.

### Polymer synthesis and characterization

**Fig. 2:**
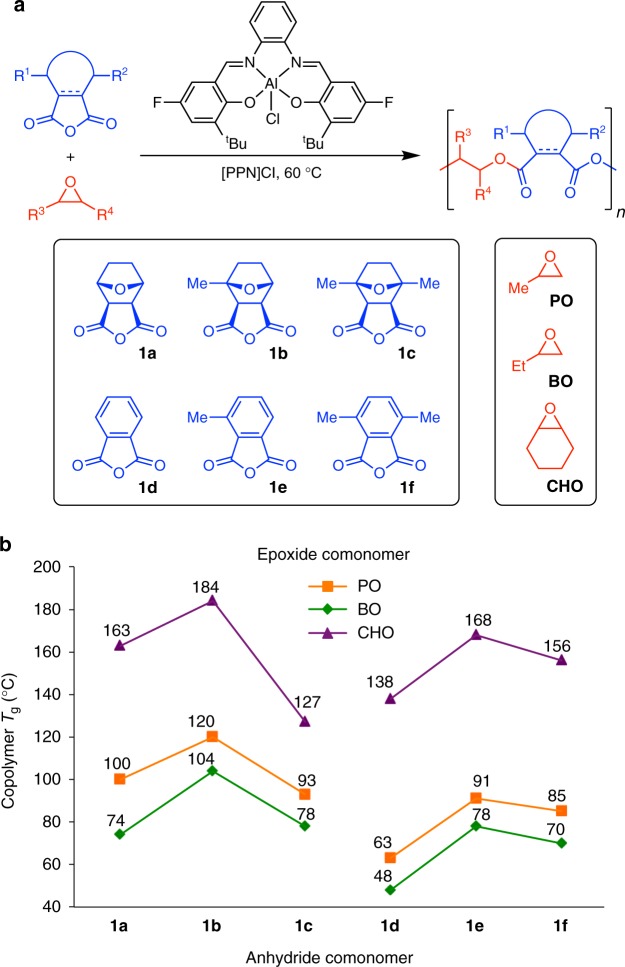
Polymer synthesis and thermal properties. **a** Cyclic anhydride and epoxide comonomers investigated in this work. **b** Observed *T*_g_ values for copolymers made from the PO, BO, and CHO epoxides and the tricyclic (**1a**–**1c**) or phthalic (**1d**–**1f**) anhydride comonomers

We then systematically investigated the copolymerization of these anhydrides using propylene oxide (PO), 1-butene oxide (BO), and cyclohexene oxide (CHO) in the presence of an aluminum catalyst^19^ (abbreviated as [^F^Salph]AlCl) and bis(triphenylphosphine)iminium chloride ([PPN]Cl), as shown in Fig. 2a (see Methods section). Their unsubstituted analogs (**1a**, **1d**) were also included in this study for comparative purposes, and it is worth noting that the copolymerization of **1d** with various epoxides has been extensively reported in the literature^20–24^. Under appropriate ratios of monomer to catalyst, 18 polyesters (Fig. 2) were prepared with well-controlled molecular weights (*M*_n_ ~20 kDa for PO and BO copolymers, *M*_n_ ~10 kDa for CHO copolymers) and narrow dispersities (Supplementary Tables 1–2). No evidence of ether linkage formation was observed by NMR spectroscopy (spectra of polymers are provided in Supplementary Figures 6–23). Differential Scanning Calorimetry (DSC) was used to measure the *T*_g_ values of all polymers (see Methods section), which are shown in Fig. 2b. It is worth noting that the *T*_g_ values of the PO/anhydride polymers remain almost unchanged with increased molecular weight (Supplementary Table 1), confirming the hypothesis that the observed differences in *T*_g_ values are due to structural variation between the polymers instead of molecular weight discrepancies between samples.

Due to their bulky tricyclic nature, polymers made from **1a–1c** are generally characterized by higher *T*_g_ values than those made from **1d**–**1f** with the same number of methyl substituents (Fig. 2b). When the epoxide was varied, we observed that the BO-based polymers have lower *T*_g_ values than the PO-based polymers and attribute this to the fact that longer alkyl groups often hinder polymer chains from densely packing^25,26^. Furthermore, the CHO-based polymers have substantially higher *T*_g_ values relative to both the PO- and BO-based analogs due to the decreased chain flexibility imposed by the cyclohexyl ring^8^. Quite interestingly, we also found that the addition of methyl substituents to the anhydride comonomers has a non-linear influence over the observed *T*_g_ values, despite the widely accepted notion that *T*_g_ values increase upon methyl substitution due to the additional steric hindrance near the polymer backbone^8,10^. In particular, we found that polyesters made from monomethyl-substituted anhydrides exhibited higher *T*_g_ values than those made from the corresponding unsubstituted as well as dimethyl-substituted anhydrides. In other words, polymers with an intermediate number of methyl substituents exhibited the highest *T*_g_ values in each series. Given the ubiquity of methyl substitution in polymer structures, we now turn to a detailed analysis of the backbone rotational flexibility in these polymers accompanied by a discussion of the key chemical and physical insights that we have gained into this unexpected structure-property relationship. We believe that these findings will aid in the rational design of synthetic polymers with a wide range of targeted thermal properties.

### Chain flexibility and glass transition temperature

In general, *T*_g_ values are governed by the overall cooperative segmental mobility^27,28^ of the polymer, which in turn is determined by its intrinsic chain (or backbone) flexibility and the non-bonded intermolecular interactions present in the system^8,29^. Since the polymers considered in this work do not exhibit strong secondary forces (e.g., there are no directional hydrogen bonding motifs like one would find in polyamides, for instance) and only differ (within a given series) by the presence of one or two methyl groups along the backbone (which will not provide substantive differences in the non-bonded dispersion interactions), we can eliminate non-bonded intermolecular interactions as being the primary driving forces responsible for this aforementioned structure-property relationship. We therefore hypothesize that intrinsic chain flexibility is predominantly responsible for the fact that the monomethyl-substituted polyesters exhibited the highest *T*_g_ values in each series. To support this claim, we present both experimental and theoretical evidence that intrinsic chain flexibility is indeed the primary difference between theses systems and that the monomethyl-substituted polymers have the lowest chain flexibility within their respective series.

To provide experimental support that the addition of a single methyl substituent leads to a substantial decrease in polymer mobility, the average molecular hole volumes (*V*_h_)^30–32^ associated with all six PO-based polyesters considered in this study were measured via positron annihilation lifetime spectroscopy (PALS)^33,34^ (Supplementary Methods), as glassy polymers with lower chain flexibility are generally accompanied with larger *V*_h_^35–37^. In doing so, we found that the monomethyl-substituted polyesters in both the tricyclic and phthalic series indeed have the largest respective *V*_h_ (Supplementary Table 3). Since the monomethyl-substituted polyesters have both larger *V*_h_ and larger *T*_g_ values (when compared with their unsubstituted and dimethyl-substituted counterparts), these findings provide a direct correlation between intrinsic chain flexibility and the anomalous *T*_g_ relationship observed in these polyesters.

### Theoretical analysis

**Fig. 3:**
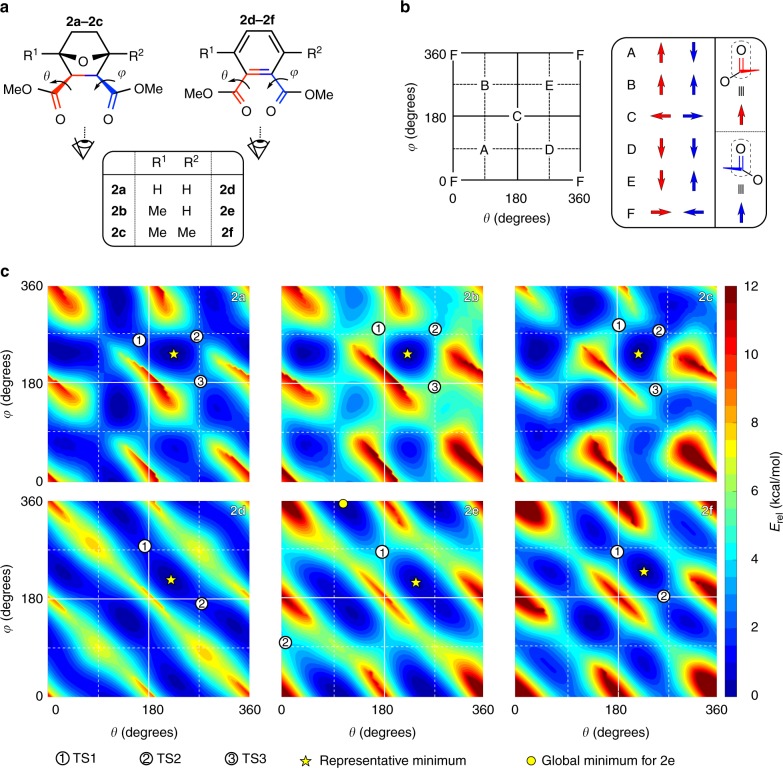
Ramachandran-type analysis. **a** Model compounds for the tricyclic (**2a**–**2c**) and phthalic (**2d**–**2f**) anhydride-based polyesters considered in this work. The bonds used to specify the *θ* and *φ* dihedral angles are delineated in red and blue, respectively, with the corresponding perspectives defined above. **b** Graphical legend illustrating the relative orientations of the two carbonyl groups corresponding to a given set of *θ* and *φ* dihedral angles. Red and blue arrows are used to depict the orientation of the left and right carbonyl groups, respectively. For example, conformation A (with dihedral angles given by *θ* = 90° and *φ* = 90°) corresponds to a structure with the left carbonyl group orientated upward and the right carbonyl group orientated downward. **c** Ramachandran-type plots depicting the full 2D PES corresponding to dihedral rotations about *θ* and *φ* for each of the polyester model compounds (**2a**–**2f**). Select stationary points on each PES include a representative minimum (gold star) and its most accessible neighboring transition states (circled numbers), the structures and energetics of which will be showcased in Fig. 5. The gold star corresponds to the location of the global minimum for all cases except **2e**, where the global minimum (with an energetic separation of ~0.36 kcal/mol) is indicated with a gold circle (Supplementary Discussion and Supplementary Table 6)

To further investigate the intrinsic chain flexibility in these methyl-substituted polyesters, we employed dispersion-inclusive hybrid density functional theory, an ab initio quantum mechanical approach that simultaneously ameliorates self-interaction error^38,39^ and accounts for nonlocal correlation effects such as dispersion (or van der Waals) interactions^40,41^. As such, this approach is able to furnish an accurate and reliable description of the rotational barriers along the polymer backbone (see Methods section, Supplementary Discussion, and Supplementary Tables 4–5). In particular, we focused our computational efforts on determining the rotational flexibility associated with the bonds between the carbonyl carbons and their respective α-carbons, which are defined by the *θ* and *φ* angles in Fig. 3a, due to their proximity to the methyl substituents introduced on the anhydride-derived repeat units (Fig. 2a). As illustrated in Fig. 2b, the non-linear influence exhibited by methyl substitution on *T*_g_ is effectively independent of the choice for the epoxide comonomer (Supplementary Discussion and Supplementary Figure 32), which further justifies our focus on these select rotational degrees of freedom as well as the computationally tractable model systems (**2a**–**2f**) employed throughout this study (Fig. 3a).

Since the two ester groups are vicinally substituted on the rings of these polyesters, there should be significant coupling between the *θ* and *φ* rotational degrees of freedom. Hence, instead of obtaining one-dimensional (1D) scans^42–44^ of the potential energy surfaces (PES) corresponding to independent dihedral rotations around *θ* and *φ*, we performed full two-dimensional (2D) scans of the rotational PES for each polyester model compound (see Methods section). This approach is analogous to the Ramachandran plots used to study the rotational flexibility of the peptide backbone in proteins^45,46^ and allows us to investigate the collective nature of these dihedral rotations. In doing so, we can provide a more detailed and comprehensive characterization of the local backbone flexibility, which we believe to be primarily responsible for the overall backbone flexibility in this class of polymers and hence the *T*_g_ trends observed herein.

Detailed Ramachandran-type plots of the 2D rotational PES for systems **2a**–**2f** are presented in Fig. 3c (with representative conformations described in Fig. 3b). Since all of these plots are diagonally dominant (i.e., the location of the minima and most accessible transition states (TS) are located along the diagonal direction), one can first conclude that dihedral rotations around *θ* and *φ* are indeed strongly coupled. In this regard, the presence of such collective rotational degrees of freedom in these polyesters is in stark contrast to the simple 1D plots that are adequate to describe internal rotations in simple molecules such as ethane, butane, or other polyesters such as polyethylene terephthalate (vide infra). In fact, independent rotations of either *θ* or *φ* are significantly hindered in all cases (as evidenced by the largest energetic barriers located along the horizontal and vertical directions in these Ramachandran-type plots) and the primary mechanism governing rotational flexibility in this class of polymers is therefore best described as collective and disrotatory motions of the vicinal ester groups.

Upon the addition of methyl substituents, the rotational landscapes of both the tricyclic and phthalic series are significantly modified, showing non-linear changes in the relative chain flexibilities in these polymers. For one, the introduction of a single methyl group breaks the left-right reflection symmetry in these systems, which lifts the degeneracies across the diagonal as well as the near-degeneracies along the diagonal. The presence of this methyl substituent also results in a reduction in the overall number of (local) minima and TS on the rotational PES corresponding to **2b** and **2e**. In fact, this addition can also lead to a completely different global minimum conformation, as observed in the phthalic anhydride series (see **2e** in Fig. 3c and Supplementary Discussion). Of particular importance is the fact that the rotational barriers are noticeably higher in the Ramachandran-type plots corresponding to both **2b** and **2e**, which is clear evidence that rotational flexibility is hindered in the monomethyl-substituted compounds. Since left-right reflection symmetry is restored upon addition of the second methyl group, such deleterious effects of symmetry breaking are completely ameliorated in the rotational PES corresponding to **2c** and **2f**, which are characterized with rotational barriers that are only slightly increased with respect to their completely unsubstituted counterparts. Although symmetry breaking (and its subsequent restoration) is the underlying reason behind these observed trends in chain flexibility, a detailed analysis of these rotational PES allows us to procure key insight into how substituent effects influence chain flexibility in these polyesters (vide infra).

**Fig. 4:**
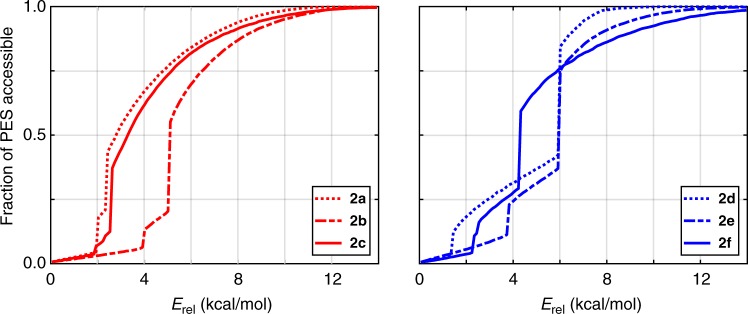
Rotational PES accessibility. Fractional area of the 2D rotational PES depicted in Fig. 3c that is accessible from the respective global minima corresponding to the tricyclic (**2a**–**2c**, left panel) and phthalic (**2d**–**2f**, right panel) model compounds as a function of the energy (*E*_rel_) available for traversing rotational barriers (Supplementary Discussion)

The modifications to the rotational landscape upon methyl substitution also lead to substantial changes in the equilibrium (thermal) population of the relevant energy minima on these PES, as well as the connectivity and energetic barriers between such stationary points. Looking again at the Ramachandran-type plots in Fig. 3c, one immediately notices that large swaths of the rotational PES are less accessible in the monomethyl-substituted polymers (**2b**, **2e**) when compared with their unsubstituted and dimethyl-substituted counterparts. As shown in Fig. 4, we quantitatively assessed this measure of rotational flexibility by plotting the fractional area of each 2D rotational PES that is accessible from the corresponding global minimum conformation as a function of the thermal energy (*E*_rel_) available for traversing potential rotational barriers (Supplementary Discussion and Supplementary Figure 33). In the tricyclic series, the accessible fractional area is considerably smaller in **2b** (when compared against **2a** and **2c**) across all values of *E*_rel_, which indicates that **2b** is the least flexible among these three structures with respect to *θ* and *φ* dihedral rotations. In the phthalic series, we also notice that the accessible fractional area is smaller in **2e** in the relevant low- and intermediate-energy sectors. In this case, the slightly larger accessible fractional area of **2e** for *E*_rel_ values in the range of 0.0–2.0 kcal/mol is inconsequential to the overall rotational flexibility of this polymer because this area corresponds to local (frustrated) rotational motion confined to the energetic basin of the global minimum (Fig. 3c and Supplementary Discussion). Quite interestingly, the relatively wide valley surrounding the global minimum in **2e** is a direct consequence of the symmetry breaking caused by the addition of a single methyl group in this polymer series (vide supra), in which the three distinct minima present in **2d** (or **2f**) are effectively merged into two wide and fairly shallow minima. We have also devised an alternative statistical mechanical measure of the rotational flexibility in these polymers that accounts for the thermal population of all points on the rotational PES, which we denote as the thermal flexibility index ($\hat F$, see Methods section). At 25 °C, we find $\hat F$ values of 11.7 (**2a**), 2.7 (**2b**), 8.2 (**2c**) and 9.9 (**2d**), 3.8 (**2e**), 6.2 (**2f**), again clearly indicating that the monomethyl-substituted cases are indeed the least flexible in both series. We note in passing that the same trend is also observed at 100 °C, which is a more relevant temperature for discussing high-*T*_g_ polymer applications. Taken together, both of these measures of rotational flexibility are consistent with our hypothesis that backbone flexibility is primarily responsible for the experimental observation that the polyesters with an intermediate number of methyl substituents exhibit the highest *T*_g_ values.

**Fig. 5:**
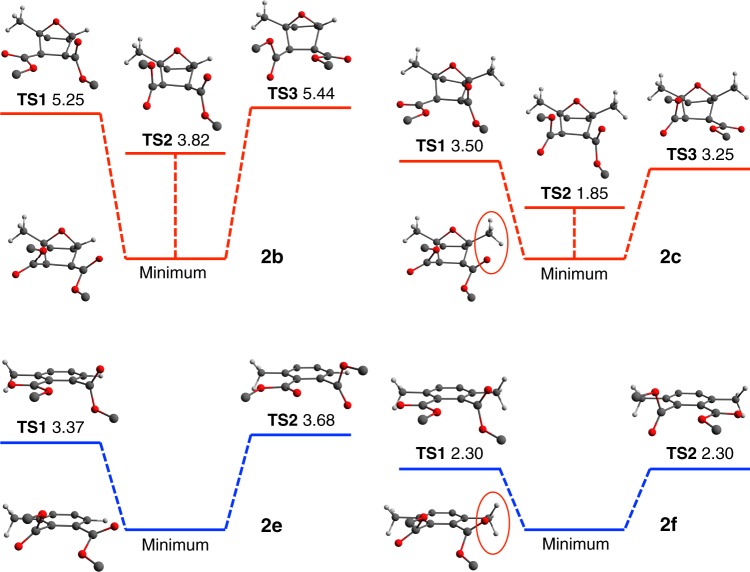
Stationary point characterization. Structures and relative energetics (in kcal/mol) of the highlighted stationary points on the 2D rotational PES depicted in Fig. 3c for the monomethyl-(**2b**, **2e**) and dimethyl-(**2c**, **2f**) substituted model compounds. The analogous barriers for the unsubstituted compounds in each series were omitted for clarity and determined to be +2.01 (**TS1**), +2.42 (**TS2**), +2.78 (**TS3**) for **2a** and +1.38 (**TS1**), +1.38 (**TS2**) for **2d**. The energetically unfavorable steric strain between ester groups and methyl substituents are indicated with red circles

In order to pinpoint the underlying cause of the substantially increased flexibility observed with the introduction of a second methyl group in compounds **2c** and **2f**, we also analyzed the structures and relative energetics associated with a representative minimum (across each series) as well as its neighboring and most accessible TS conformations (Fig. 5). The locations of these stationary points on the Ramachandran-type plots are highlighted in Fig. 3c, from which one can see that the paths connecting any one of these minima and its corresponding TS conformations involves concerted rotations along *θ* and *φ*. This again stresses the collective nature of these rotational degrees of freedom and the utility of the Ramachandran-type analysis performed herein. In both the tricyclic and phthalic series, the relative energetics corresponding to the TS structures in Fig. 5 again confirm that all rotational barriers are indeed lower in the dimethyl-substituted compounds, which provides additional support to our hypothesis that the addition of a second methyl group leads to increased rotational flexibility in these polymers.

What is not immediately clear from the energetics in Fig. 5 is that this trend is not due to relative stabilization of the TS conformations in **2c** (or **2f**) and instead results from relative destabilization of the dimethyl-substituted minima. In this regard, we note that both the representative minimum and the TS have very similar conformations when going from **2b** to **2c** (and **2e** to **2f**), yet the addition of a second methyl group introduces significant additional strain into the system from the perspective of the minima, as depicted in Fig. 5. This observation indicates that steric interactions between the vicinal ester groups are the predominant source of strain in these systems, a key finding that is also supported by the significant coupling found between these rotational degrees of freedom even in the absence of methyl substituents (**2a** and **2d** in Fig. 3c). In each representative minimum, we believe that the steric strain between the vicinal ester groups is ameliorated by the favorable Bürgi-Dunitz angle^47^ between the left alkoxy oxygen donor and the right carbonyl carbon acceptor, which allows for favorable orbital overlap. Upon the introduction of either one or two methyl groups, the system must minimize the steric interactions between the strongly coupled vicinal ester groups, whose motions are now further constrained by the presence of methyl-induced strain. Hence, the system adopts the optimal rotational configuration for the ester groups in the minimum energy conformations in **2b** and **2c** (**2e** and **2f**), despite the additional methyl-induced strain present in the dimethyl-substituted compounds. In the corresponding TS conformations, the fact that the ester groups are specifically not in their optimal rotational configuration essentially governs their relative energetic profiles and overshadows any potentially unfavorable steric interactions with the methyl groups. These findings are further supported by a detailed analysis of a series of intramolecular symmetry-adapted perturbation theory^48–50^ calculations that were used to further investigate this complex interplay between ester–ester steric interactions and methyl-induced strain (Supplementary Discussion, Supplementary Table 7, and Supplementary Figure 34). Since the additional methyl-induced strain that is present in the dimethyl-substituted compounds has a significantly larger impact on the relative stabilities of the minima rather than the TS conformations, the net effect is a relative destabilization of the minimum energy conformations in **2c** and **2f**. This results in the lower apparent rotational barrier heights seen in Figs. 3c and 5, which in turn correspond to significant relative increases in the flexibility of these polymer chains with the introduction of a second methyl substituent.

## Discussion

From this work, one sees clear experimental evidence that selective methyl substitution can be leveraged to tune the observed *T*_g_ values in polyesters with strongly coupled vicinal ester groups. Since none of these polyesters are subject to strong secondary forces and only differ by the presence of one or two methyl groups along the backbone within a given series, we eliminate non-bonded intermolecular interactions as being the primary driving forces responsible for this aforementioned structure–property relationship. This claim is further supported experimentally by the fact that the monomethyl-substituted polyesters in both the tricyclic and phthalic series indeed have the largest respective *V*_h_ within a given series, thereby providing a direct correlation between chain flexibility and the anomalous *T*_g_ relationship observed in these polyesters.

These experimental findings are also accompanied by a detailed theoretical analysis of the nonlinear influence of these methyl substituents on the backbone rotational flexibility of these polyesters. In doing so, we demonstrate that the underlying quantum mechanical mechanism responsible for this trend is the compromise made by the system in optimizing the rotational configuration of the strongly coupled vicinal ester groups (which are the primary source of strain in these systems) in the presence of methyl-induced strain. A key finding uncovered here is that this results in a relative destabilization of the minimum energy conformations in the dimethyl-substituted cases, which in turn lead to lower apparent rotational barriers and (by extension) increased chain flexibility. As such, the analysis presented herein provides key chemical and physical insight into how substituent effects influence intrinsic polymer chain flexibility, and takes us one step closer to the rational (and even computational^51^) design of high-*T*_g_ polymers.

Quite interestingly, these findings are not only limited to the class of polyesters with strongly coupled vicinal ester groups, and can be used rather straightforwardly to rationalize the reported^52^ *T*_g_ differences between poly(ethylene terephthalate) and poly(ethylene methyl-terephthalate), in which the rotational motions of the quite distant ester groups are completely independent (Supplementary Discussion and Supplementary Figure 35). This example is strongly indicative that our findings are robust and will provide key insight into the relationship between structure and thermal properties across a range of synthetic polymers.

## Methods

### General experimental details

All manipulations of air- and water-sensitive compounds were carried out under nitrogen in a MBraun Labmaster glovebox or by using standard Schlenk line techniques. Epoxides were purchased from Aldrich and distilled from calcium hydride. Bis(triphenylphosphine)iminium chloride ([PPN]Cl) was also purchased from Aldrich and recrystallized by layering a saturated methylene chloride solution with diethyl ether. Phthalic anhydride was purchased from Aldrich and recrystallized from hot chloroform. The synthesis of all other anhydrides and the [^F^Salph]AlCl catalyst is detailed in Supplementary Methods and NMR spectra of these anhydrides are provided in Supplementary Figures 1–5.

### Copolymerization procedure

In a glovebox, the appropriate amount of [^F^Salph]AlCl catalyst (1.1–4.3 µmol, depending on the anhydride to catalyst ratio) and [PPN]Cl (1.0–3.9 µmol, 0.9 equivalents relative to the catalyst) were placed in an oven-dried 4-mL vial equipped with a magnetic stir bar. Cyclic anhydride (1.3 mmol) was added, followed by the addition of a predetermined amount of epoxide (and solvent) depending on the epoxide choice (Supplementary Methods). The vial was sealed with a Teflon-lined cap, removed from the glovebox, and placed in an aluminum heating block preheated to 60 °C. After the appropriate amount of time, an aliquot was taken for ^1^H NMR spectroscopic analysis to determine conversion of the cyclic anhydride. The reaction mixture was then diluted with ~0.5 mL methylene chloride and precipitated into 10 mL of methanol with vigorous stirring, after which the methanol was decanted. Polymers made from **1a**–**1c** were precipitated into hexanes due to their higher solubility in methanol. Precipitation was repeated as necessary to remove excess monomer and catalyst. The polymer was then dried under vacuum at 60 °C for 3 days and used for physical measurements. Synthetic and characterization details are provided in Supplementary Tables 1–2 and NMR spectra of all polymers are provided in Supplementary Figures 6–23.

### DSC measurements

DSC measurements of the polymer samples were performed on a Mettler-Toledo Polymer DSC instrument equipped with a chiller and an autosampler. Samples were prepared in aluminum pans. All polyesters were analyzed using the following temperature program: −70 to 200 °C at 10 °C/min, 200 to −70 °C at 10 °C/min, and then −70 to 200 °C at 10 °C/min. Data were processed using the StarE software. All reported *T*_g_ values were observed on the second heating cycle. All DSC thermograms are provided in Supplementary Figures 24–31.

### Computational details

Each of the Ramachandran-type plots in Fig. 3c was constructed from the energies corresponding to 72 × 72 constrained geometry optimizations (for a total of 5184 calculations per model compound, taken in increments of 5° along the collective variable coordinates defined by *θ* and *φ*). The accompanying periodic surface plots (heat maps) were created using bilinear interpolation of the data with an in-house script written in the Matlab 2017b program. All geometry optimizations were performed (using the corresponding default convergence thresholds) with the Q-Chem (version 5.0) software package^53^. Unless otherwise specified, all calculations employed the B3LYP hybrid exchange-correlation functional^54–56^ in conjunction with the D3(op) dispersion correction^57,58^ and the 6–311++ G(d,p) basis set. This level of theory was selectively tested against higher-level quantum chemical methods (such as CCSD) with excellent performance reported for characterizing internal bond rotations in the systems considered in this work (Supplementary Discussion and Supplementary Tables 4–5). The optimized geometries and harmonic vibrational frequencies for all stationary points reported in Fig. 5 were obtained using the tightest convergence thresholds available in Q-Chem to accurately reflect the structures and energetics of the true stationary points on the global PES. For a select set of stationary point structures, intramolecular symmetry-adapted perturbation theory^50^ (SAPT) calculations were performed at the density-fitted SAPT0 (DF-SAPT0) level using the PSI4 (version 1.1) software package^59^, with the jun-cc-pVDZ basis set and the corresponding JKFIT and MP2FIT auxiliary basis sets. All core orbitals were frozen during the SAPT calculations.

### Thermal flexibility index

In order to introduce the thermal flexibility index ($\hat F$), we first define the thermal flexibility (*F*), which is an ensemble average over all conformational transitions on the rotational PES in Fig. 3c, as follows:

1 $$F = {\int} {{\mathrm{d\alpha }}{\int} {{\mathrm{d\beta }}\rho ^ \ast \left( {\mathrm{\beta }} \right){\mathrm{e}}^{ - E^ \ne \left( {{\mathrm{\alpha }} \leftarrow {\mathrm{\beta }}} \right)/k_BT} \approx \frac{A}{N}\mathop {\sum}\limits_\alpha {\mathop {\sum}\limits_\beta {\frac{{{\mathrm{e}}^{ - E_\beta /k_BT}}}{{\mathop {\sum}\nolimits_\gamma {{\mathrm{e}}^{ - E_\gamma /k_BT}} }}{\mathrm{e}}^{ - E^ \ne \left( {{\mathrm{\alpha }} \leftarrow {\mathrm{\beta }}} \right)/k_BT}} .} } }$$

In this expression, *ρ*^∗^(*β*) denotes the probability density associated with conformation *β* (given by a Boltzmann distribution at temperature *T*), $E^ \ne \left( {{\mathrm{\alpha }} \leftarrow {\mathrm{\beta }}} \right)$ represents the maximum barrier height along the minimum energy path connecting conformations *α* and *β*, and *A* is the total area of the rotational PES (that has been discretized by *N* points). In other words, *F* is the Boltzmann factor from transition state theory applied to the barriers encountered during conformational transitions, which has been thermally averaged and summed over all possible transitions. In this work, a modified version of the Floyd-Warshall algorithm^60–62^ was employed to compute $E^ \ne \left( {{\mathrm{\alpha }} \leftarrow {\mathrm{\beta }}} \right)$ between any two conformations on a given rotational PES. In particular, instead of evaluating the length of a combined path by addition of two subpath lengths (as was done in the original Floyd-Warshall algorithm), we evaluate $E^ \ne \left( {{\mathrm{\alpha }} \leftarrow {\mathrm{\beta }}} \right)$ for the combined path by choosing the higher maximum barrier of the two subpaths. Since the thermal flexibility is maximized for a (fictitious) completely flat rotational PES, in which the probability density is uniform and $E^ \ne \left( {{\mathrm{\alpha }} \leftarrow {\mathrm{\beta }}} \right) = 0$ for any two conformations, we use this quantity ($F_0 = {\int} {{\mathrm{d\alpha }} = A}$) as a reference for defining $\hat F$. In doing so, the $\hat F$ corresponding to a given PES is defined as the ratio $\hat F = F/F_0 \times 100$ (such that $\hat F$ is always between 0 and 100), which allows for direct comparison of thermal flexibility indices corresponding to different PES.

### Data availability

All data are available from the authors upon reasonable request.

## Electronic supplementary material

Supplementary Information
