# Supplementary material for: Unraveling substituent effects on the glass transition temperatures of biorenewable polyesters
Source: Nat Commun. 2018 Jul 23;9:2880. doi: 10.1038/s41467-018-05269-3 (PMC6056526; doi:10.1038/s41467-018-05269-3)
Supplement: Supplementary file 1 — Supplementary Information [file 41467_2018_5269_MOESM1_ESM.pdf]

Supplementary Information for

Unraveling Substituent Effects on the Glass Transition Temperatures of Biorenewable Polyesters

Xiaopeng Yu,[1,#] Junteng Jia,[1,#] Shu Xu,[2] Ka Un Lao,[1] Maria J. Sanford,[1] Ramesh K. Ramakrishnan,[3] Sergei I. Nazarenko,[3] Thomas R. Hoye,[2] Geoffrey W. Coates,[1,*] and Robert A. DiStasio Jr.[1,*]

[1]*Department of Chemistry and Chemical Biology, Cornell University, Ithaca, NY 14853, USA*

[2]*Department of Chemistry, University of Minnesota, Minneapolis, MN 55455, USA*

[3]*School of Polymers and High Performance Materials, University of Southern Mississippi, Hattiesburg, MS 39402, USA*

[#]These authors contributed equally to this work.

[*]Corresponding authors: coates@cornell.edu; distasio@cornell.edu

Supplementary Methods

Instrument. ^{1}H NMR spectra were recorded on a Bruker AV III HD spectrometer with a broadband Prodigy CryoProbe (^{1}H, 500 MHz) and referenced to the residual chloroform (7.26 ppm) signals. ^{13}C NMR spectra were recorded on a Bruker AV III HD spectrometer with a broadband Prodigy CryoProbe (^{13}C, 126 MHz) spectrometer and referenced to the residual chloroform (77.16 ppm) signals. DART-HRMS analyses were performed on a Thermo Scientific Exactive Orbitrap MS system equipped with an Ion Sense DART ion source. Gel permeation chromatography (GPC) analyses were carried out using an Agilent 1260 Infinity GPC System equipped with a refractive index detector, an Agilent 1260 Infinity autosampler, and two Agilent PolyPore columns (5 micron, 4.6 mm ID) which were eluted with THF at 30 °C at 0.3 mL/min and calibrated using monodisperse polystyrene standards.

Materials. Solvents used for cyclic anhydride and ligand synthesis, including methanol (Macron), absolute ethanol (Koptec), methylene chloride (Fisher), hexanes (Macron), ethyl acetate (Fisher), chloroform (Fisher), and diethyl ether (J. T. Baker), were used as received. *Exo*-3,6-epoxy-1,2,3,6-tetrahydrophthalic anhydride (Aldrich), 2-methylfuran (TCI, > 98%), 2,5-dimethylfuran (Aldrich, 99%), and maleic anhydride (Aldrich, ≥ 99.0%) were used as received. Hydrogen (Airgas, 99.99%) was used as received. [^{F}Salph]AlCl was synthesized according to a literature procedure[1]. All other chemicals and reagents were purchased from commercial sources (Aldrich, Combi-Blocks, Strem, Acros, TCI, and Alfa Aesar) and used without further purification.

Synthesis of Cyclic Anhydrides

***cis*-*exo*-7-Oxabicyclo[2.2.1]heptane-2,3-dicarboxylic anhydride (1a).** The following procedure was adapted from the literature[2]: In a 250 mL Parr reactor, *exo*-3,6-epoxy-1,2,3,6-tetrahydrophthalic anhydride (5.00 g, 30.1 mmol) was dissolved in ethyl acetate (140 mL), and palladium on carbon (5 wt. %, 1.00 g) was added. The reactor was purged with nitrogen then pressurized with 650 psi hydrogen gas. After stirring at 22 °C for 12 h, the reactor was carefully vented, and the crude reaction mixture was filtered through a pad of silica gel (10 g). The solvent was removed and the residue was dried under vacuum overnight to provide **1a** as a white solid (4.90 g, 97%). ^{1}H NMR (500 MHz, $CDCl_3$): δ 4.99 (dd, J = 3.4, 2.2 Hz, 2H), 3.18 (s, 2H), 1.85 (m, 2H), 1.63 (dt, J = 14.1, 3.4 Hz, 2H). ^{13}C NMR (126 MHz, $CDCl_3$): δ 171.49, 80.26, 50.66, 28.16. HRMS (DART-MS): *m/z* calculated for $C_8H_9O_4$ (M+H) 169.0501, found 169.0492.

***cis*-*exo*-1-Methyl-7-oxabicyclo[2.2.1]heptane-2,3-dicarboxylic anhydride (1b).** The following procedure was adapted from the literature[3]: Maleic anhydride (2.74 g, 27.8 mmol) was mixed with 2-methylfuran (2.28 g, 27.8 mmol). The resulting mixture was stirred at ambient temperature for 17 h. The solidified reaction mixture was used in the next step without further purification. A portion of this solidified Diels-Alder adduct (4.25 g, 23.6 mmol) was dissolved in acetone (40 mL) in a 150 mL two-neck round-bottom flask. Palladium on carbon (10 wt. %, 400 mg) was added and the reaction mixture was sparged with hydrogen gas for 3 h under stirring. The resulting suspension was filtered through a pad of Celite, which was washed thoroughly with acetone (20 mL). The combined filtrate was concentrated *in vacuo* to provide an off-white solid, which was dried overnight under vacuum to give the crude product. This crude product was purified by flash column chromatography on silica gel using 25% ethyl acetate in hexanes as

the eluent and then dried under vacuum overnight to provide **1b** as white solid (3.87 g, 90%). ^1H NMR (500 MHz, $CDCl_3$): δ 4.90 (d, *J* = 5.2 Hz, 1H), 3.26 (d, *J* = 7.4 Hz, 1H), 3.05 (d, *J* = 7.4 Hz, 1H), 2.00 (m, 1H), 1.69 (m, 3H), and 1.63 (s, 3H). ^{13}C NMR (126 MHz, $CDCl_3$): δ 171.70, 170.07, 86.94, 79.79, 52.80, 52.34, 35.71, 30.01, 17.59. HRMS (DART-MS): *m/z* calculated for $C_9H_{11}O_4$ (M+H) 183.0657, found 183.0649.

***cis-exo*-1,4-Dimethyl-7-oxabicyclo[2.2.1]heptane-2,3-dicarboxylic anhydride (1c).** This compound was prepared according to a literature procedure[4]. ^1H NMR (500 MHz, $CDCl_3$): δ 3.14 (s, 2 H), 1.78 (m, 4 H), 1.60 (s, 6H). ^{13}C NMR (126 MHz, $CDCl_3$): δ 170.17, 86.08, 54.40, 37.65, 17.91. HRMS (DART-MS): *m/z* calculated for $C_{10}H_{13}O_4$ (M+H) 197.0808, found 197.0801.

3-Methylphthalic anhydride (1e). The following procedure was adapted from the literature[5]: Maleic anhydride (2.74 g, 27.8 mmol) was mixed with 2-methylfuran (2.28 g, 27.8 mmol). The resulting mixture was stirred at ambient temperature for 17 h. The solidified reaction mixture was used in the next step without further purification. A portion of this solidified Diels-Alder adduct (1.00 g, 5.56 mmol) was suspended in chloroform (10 mL) in a 50 mL two-neck round-bottom flask. Trifluoromethanesulfonic acid (40 µL, 0.45 mmol) was added. A brown-black color was immediately observed. This solution was heated to 80 °C at which time the color became somewhat darker and a black solid material precipitated. The chloroform supernatant was decanted and the black residue was washed with a portion of fresh chloroform. The combined chloroform layers were concentrated *in vacuo*. The residue was purified by flash column chromatography on silica gel using 25% ethyl acetate in hexanes as the eluent and then dried over vacuum overnight to give **1e** (0.55 g, 60%) as white crystalline solid. ^1H NMR (500 MHz, $CDCl_3$): δ 7.82 (d, *J* = 7.6 Hz, 1H), 7.76 (t, *J* = 7.6 Hz, 1H), 7.66 (d, *J* = 7.6 Hz, 1H), 2.72 (s, 3H). ^{13}C NMR

(126 MHz, $CDCl_3$): δ 163.20, 163.10, 140.57, 138.06, 135.83, 131.66, 128.56, 123.38, 17.79. HRMS (DART-MS): *m/z* calculated for $C_9H_7O_3$ (M+H) 163.0395, found 163.0387.

3,6-Dimethylphthalic anhydride (1f). The following procedure was adapted from the literature[6]: Maleic anhydride (2.74 g, 27.8 mmol) was mixed with 2,5-dimethylfuran (2.67 g, 27.8 mmol). The resulting mixture was stirred at ambient temperature for 17 h. The solidified reaction mixture was used in the next step without further purification. A portion of this solidified Diels-Alder (D-A) adduct (2.07 g, 10.7 mmol) was added in small portions to concentrated sulfuric acid (20 ml) cooled at -6 °C. The mixture was stirred vigorously and the temperature was not allowed to rise above 0 °C. After all the D-A adduct was added and the solution turned orange in color, the reaction mixture was allowed to slowly warm to 10 °C and was poured slowly onto crushed ice (200 g). The resulting white crystals were collected on a Buchner funnel and washed with ice water. The crystals were then dissolved in an aqueous solution of sodium hydroxide (1.5 g in 15 mL H_2O). Glacial acetic acid (2.5 ml) was added to the solution. The solution was filtered to remove the insoluble materials and was then acidified with concentrated hydrochloric acid (1.5 ml). The resulting precipitant was collected and washed with water until neutral. The precipitant was recrystallized from benzene and then dried under vacuum overnight to provide the anhydride **1f** as light-yellow needles (0.90 g, 48%). ^1H NMR (500 MHz, $CDCl_3$): δ 7.50 (s, 2H), 2.66 (s, 6H). ^{13}C NMR (126 MHz, $CDCl_3$): δ 163.36, 137.90, 137.89, 128.58, 17.46. HRMS (DART-MS): *m/z* calculated for $C_{10}H_9O_3$ (M+H) 177.0552, found 177.0542.

Detailed Copolymerization Procedures

Copolymerization of propylene oxide and 1-butene oxide with cyclic anhydrides. In a glovebox, the appropriate amount of [^{F}Salph]AlCl (1.1-4.3 μmol, depending on the anhydride to cata-

lyst ratio, see Supplementary Tables 1-2 for details) and [PPN]Cl (1.0-3.9 μmol, 0.9 equivalents relative to the catalyst) were placed in an oven-dried 4-mL vial equipped with a magnetic stir bar. The appropriate amount of cyclic anhydride (1.3 mmol) was added, followed by propylene oxide (0.45 mL, 6.4 mmol) or 1-butene oxide (0.55 mL, 6.4 mmol). The vial was sealed with a Teflon-lined cap, removed from the glovebox, and placed in an aluminum heating block preheated to 60 °C. After the appropriate amount of time, an aliquot was taken for ^{1}H NMR spectroscopic analysis to determine conversion of the cyclic anhydride. The reaction mixture was then diluted with approximately 0.5 mL methylene chloride and precipitated into 10 mL of methanol with vigorous stirring, after which the methanol was decanted. Polymers made from **1a**–**1c** were precipitated into hexanes due to their higher solubility in methanol. Precipitation was repeated as necessary to remove excess monomer and catalyst. The polymer was dried under vacuum at 60 °C for three days and used for physical measurements.

Copolymerization of cyclohexene oxide with cyclic anhydrides. In a glovebox, the appropriate amount of [^{F}Salph]AlCl (2.3 mg, 4.3 μmol) and [PPN]Cl (2.2 mg, 3.9 μmol) were placed in an oven-dried 4-mL vial equipped with a magnetic stir bar. The appropriate amount of cyclic anhydride (1.3 mmol) was added, followed by cyclohexene oxide (0.39 mL, 3.9 mmol) and dry, degassed toluene (0.2 mL). The vial was sealed with a Teflon-lined cap, removed from the glovebox, and placed in an aluminum heating block preheated to 60 °C. After the appropriate amount of time, an aliquot was taken for ^{1}H NMR spectroscopic analysis to determine conversion of the cyclic anhydride. The reaction mixture was then diluted with approximately 0.5 mL methylene chloride and precipitated into 10 mL of methanol with vigorous stirring, after which the methanol was decanted. Polymers made from **1a**–**1c** were precipitated into hexanes due to their higher solubility in methanol. Precipitation was repeated as necessary to remove excess monomer and

catalyst. The polymer was dried under vacuum at 60 °C for three days and used for physical measurements.

Positron Annihilation Lifetime Spectroscopy (PALS) Experiment. Average molecular hole volume, $\langle V_h \rangle$, was probed by positron annihilation lifetime spectroscopy (PALS). The data, which corresponds to approximately 10^6 incidences for each sample, were collected over one hour at 23 °C using an Ortec Positron Lifetime System (Advanced Measurement Technology, Oak Ridge, TN) which employs a fast-fast coincidence scheme. A 30 μCi ^{22}Na positron source was sandwiched between two 1 mm thick polymer disks. The sample-source assembly was placed between two photomultiplier tubes (PMT) each equipped with BaF_2 gamma radiation sensitive scintillators and tuned so one PMT is able to differentiate and convert into a signal the absorbed gamma quanta associated with positron emission, and another PMT with positron annihilation. A multichannel analyzer compiled the coincidences resulting in determining for each positron the time difference between 'birth' and 'death' events (the lifetime) with a time resolution of 290 ps, and PATFIT-88 software analyzed the annihilation spectra assuming three components. The characteristic lifetime, τ_3, of the orthopositronium (o-Ps) annihilation component acquired from PALS spectra was first related to the average spherical hole radius, $\langle R_h \rangle$, via the Tao-Eldrup equation[7,8] and then to the average hole volume $\langle V_h \rangle = 4\pi \langle R_h \rangle^3/3$ (Supplementary Table 3).

Supplementary Discussion

Validation of computational approach. In order to justify the level of theory employed to characterize the rotational PES in this work, we compared our findings against several highly accurate quantum chemistry methods such as MP2 and CCSD. In particular, we compared the energy gap between the global minimum and the most accessible TS structure in each monomethyl-substituted compound in the tricyclic and phthalic series (**2b** in Supplementary Table 4, **2e** in Supplementary Table 5). From these results, we observed that the computed energetic gaps with B3LYP+D3(op)/6-311++G(d,p) and the most accurate method (CCSD/cc-pVTZ) are in excellent agreement (with errors less than 0.25 kcal/mol). All MP2 and CCSD single-point energy evaluations were performed with the Aquarius software package using 2048 compute nodes (each of which has 64 GB of memory and consists of 24 cores running at 2.4GHz) on Edison. The convergence criteria was set to 10^{-8} a.u. for both SCF and CCSD calculations[9].

Collective rotational degrees of freedom. In determining the intrinsic chain flexibility in this set of polyesters, one sees that there are five rotational degrees of freedom (α, β, γ, θ, φ) that govern this quantity (Supplementary Fig. 32). However, the non-linear influence exhibited by methyl substitution on T_g is independent of the choice for the epoxide comonomer (Fig. 2b), which determines the identity of R^3 and R^4 in these systems. Since one of these epoxide comonomers is cyclohexene oxide (CHO), a rigid ring structure that will completely eliminate rotational motion around α and significantly hinder rotational motion around β, these dihedral angles can be eliminated as governing the intrinsic chain flexibility in the CHO-based polyester series. Furthermore, the γ dihedral angle can also be removed from consideration as conjugation inherent to an ester functional group essentially renders it into the planar (and s-*trans*) configuration. As a

result, we conclude that rotations around the θ and φ dihedral angles—which are the only common feature among all of the polyesters considered in this work—are indeed the degrees of freedom (or collective coordinates) of primary importance, *i.e.*, it is these selective degrees of freedom that locally govern the overall chain flexibility in these polymers. Throughout this manuscript, our efforts are therefore focused on understanding how selective methyl substituents (due to their proximity to these key dihedral angles) affect collective rotations about θ and φ. In doing so, we provide extensive theoretical support that the strong coupling between the vicinal ester groups in these polymers is in fact the primary source of steric strain in these systems, and it is collective motion around θ and φ that governs local chain flexibility in this specific class of polyesters. We note here that quantitative predictions of T_g would require further investigation into how local collective rotations around θ and φ on one monomer are coupled to local collective rotations around θ' and φ' on another monomer, thereby strengthening the connection between *local* chain flexibility and overall cooperative segmental mobility in these systems.

Structural properties of the global minima. The collective variables θ and φ for the global minimum conformations of **2a-2f** are provided in Supplementary Table 6.

Accessible area on the rotational PES. An alternative way to visualize the results in Fig. 4 of the main text is to plot the area on the rotational PES that is accessible from the global minimum conformation as a function of the relative energy (E_{rel}) available for traversing rotational barriers (Supplementary Fig. 33). From this figure, one immediately sees that the monomethyl-substituted **2b** and **2e** structures are trapped in the neighborhood of their respective global minimum conformations while their unsubstituted and dimethyl-substituted counterparts can freely

explore a much larger fraction of the rotational PES. As discussed extensively in the main text, this leads to a marked decrease in the intrinsic chain flexibility in **2b** and **2e**.

Quantifying intramolecular strain. In this work, we performed a series of intramolecular symmetry-adapted perturbation theory (SAPT) calculations to characterize the steric interactions between: (1) the vicinal ester groups, (2) the ester and methyl groups on the left, and (3) the ester and methyl groups on the right (Supplementary Fig. 34). Since the steric interactions among the ester and methyl groups also cause deformation of the tricyclic and phthalic rings, we also computed the relative energies associated with the strained rings. In particular, we first optimized the geometries of the unsubstituted 7-oxabicyclo[2.2.1]heptane and benzene rings at the B3LYP+D3(op)/6-311++G(d,p) level of theory to obtain the energy of the fully relaxed rings. Then we took the geometry in each conformation, removed the ester and methyl groups on the ring, and capped the rings with hydrogens, which provides us with the deformed ring geometries. Finally, the relative ring strain energy (E_S) in a given conformation is the energetic difference between the deformed and fully relaxed ring geometries. For each structure in Supplementary Table 7, the steric strain between the vicinal ester groups (E_M) and the relative ring strain (E_S) are lower in the minimum (MIN) conformation when compared to its neighboring TSx conformations, as highlighted in green. Furthermore, when comparing the total strain energy (E_{tot}) between **2a** and **2b** (**2d** and **2e**), we note that the first methyl substitution destabilizes the TSx conformations more than the MIN conformation, as highlighted in red for ΔE. However, upon addition of the second methyl group, we observed significantly higher additional strain in the MIN rather than the TSx conformations, as shown for **2b** and **2c** (**2e** and **2f**), and highlighted in red for ΔE. In summary, this analysis demonstrates that the rotational barriers in these cases results from

a relative destabilization of the minima (and not a relative stabilization of the neighboring TS*x* conformations) in **2c** and **2f**.

Substituent effects on the T_g of poly(ethylene terephthalate). Quite interesting is the fact that we can also use this observation (that lower apparent barriers in sterically hindered polyester compounds can result from relative destabilization of the minimum energy conformations) to rationalize the reported T_g differences between poly(ethylene terephthalate) and poly(ethylene methyl-terephthalate), or PET and PMET. In these cases, we first note that the rotational motions of the ester groups in these compounds are completely independent, which is in stark contrast to compounds **2a**-**2f**, and allows us to consider the simple 1D rotational PES shown in Supplementary Fig. 35. In doing so, we see that the rotational barrier is reduced by approximately 2.3 kcal/mol upon the addition of a single methyl substituent at the B3LYP+D3(op)/6-311++G(d,p) level of theory. This trend can easily be understood by analyzing the influence of the methyl group on the global minimum ($\theta = 0°$), in which the ester group is planar with the aromatic ring to maximize conjugation, versus the transition state ($\theta = 90°$), in which the ester group is orthogonal to the aromatic ring. In this system, the introduction of a methyl substituent in the *ortho* position again leads to a relative destabilization of the ground state due to the presence of repulsive 1,3-allylic strain[10,11] between the carbonyl oxygen and the methyl group. However, this effect is minimal in the out-of-plane transition state, where the carbonyl oxygen and the methyl group are in a staggered conformation. This relative destabilization of the global minimum in PEMT leads to an apparent decrease in the rotational barrier (and therefore an increase in the intrinsic chain flexibility), which is completely consistent with the fact that PEMT (60 °C) has a lower T_g value than PET (80 °C)[12].

Supplementary Tables

Supplementary Table 1 | Copolymerization of **1a-1f** with Propylene Oxide (PO)[a]

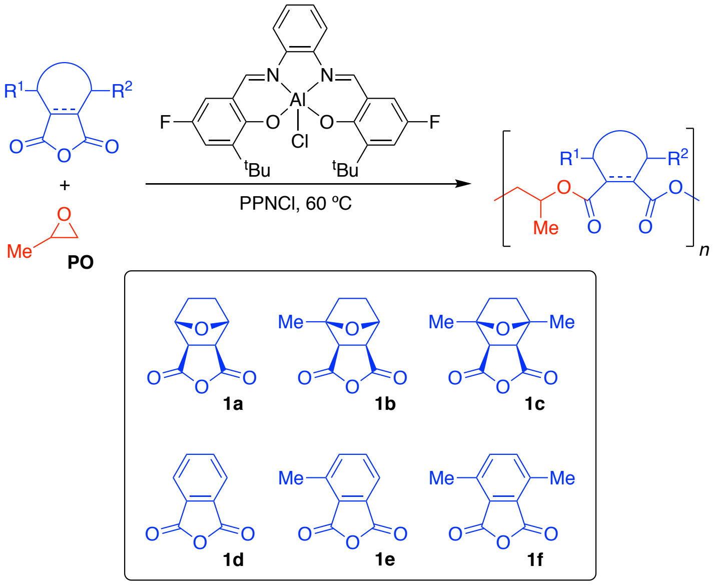

**entry**	**anhydride**	**[1]/ [Al cat]**	**t_{rxn} (h)**	**conv. of anh. (%)**[b]	**M_n (kDa)**[c]	**M_w/M_n**[c]	**T_g (°C)**[d]
1	**1a**	300	5	>99	13.8	1.12	99
2	**1b**	300	3	>99	11.8	1.15	119
3	**1c**	300	4	>99	10.7	1.15	93
4	**1a**	1200	29	>99	23.0	1.21	100
5	**1b**	1200	19	>99	32.0	1.22	120
6	**1c**	1200	24	>99	28.9	1.24	93
7	**1d**	300	3	>99	20.0	1.21	63
8	**1e**	300	2	>99	19.0	1.09	91
9	**1f**	300	2	>99	17.0	1.07	85
10	**1d**	1200	17	>99	60.9	1.33	65
11	**1e**	1200	20	>99	43.3	1.23	92
12	**1f**	1200	19	>99	34.1	1.23	87

[a]Reactions were performed in the presence of 1.3 mmol anhydride and excess neat PO (0.45 mL, 6.4 mmol). For all entries, [Al cat]:[PPNCl] = 1:0.9. [b]Conversion of anhydride determined by ^{1}H NMR spectroscopy. [c]Determined by GPC in THF at 30 °C, calibrated with polystyrene standards. [d]Determined by DSC; reported T_g values are from the second heat.

Supplementary Table 2 | Copolymerization of **1a–1f** with Butene Oxide (BO) and Cyclohexene Oxide (CHO)[a]

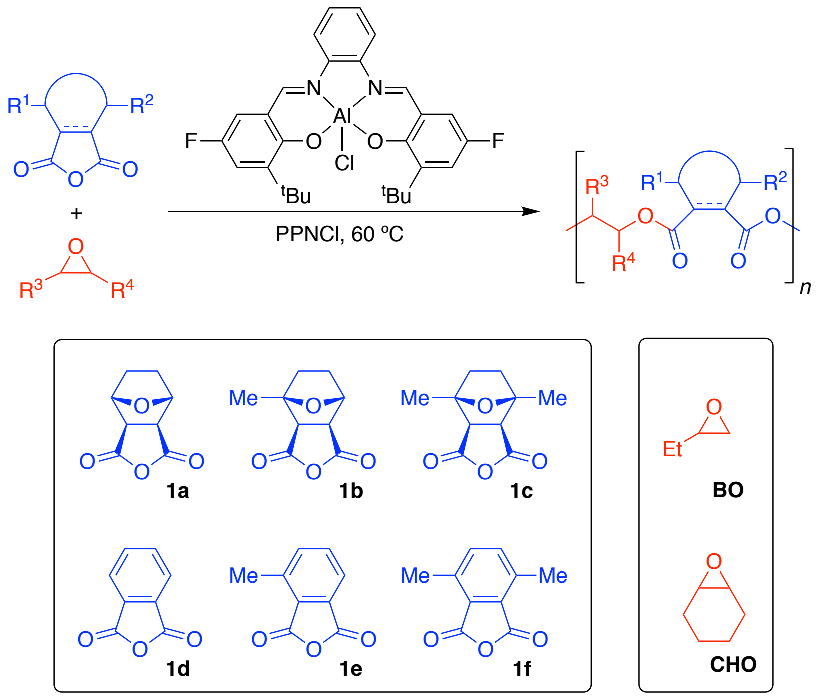

entry	epoxide	anhydride	[1]/ [Al cat]	t_{rxn} (h)	conv. of anh. (%)[b]	M_n (kDa)[c]	M_w/M_n[c]	T_g (°C)[d]
1	BO	**1a**	1200	29	>99	27.1	1.23	74
2	BO	**1b**	1200	17	>99	24.8	1.23	104
3	BO	**1c**	1200	36	>99	20.4	1.13	78
4	BO	**1d**	300	3	>99	17.0	1.23	48
5	BO	**1e**	300	3	>99	16.9	1.17	78
6	BO	**1f**	300	3	>99	14.4	1.16	70
7	CHO	**1a**	300	27	>99	10.9	1.29	163
8	CHO	**1b**	300	17	>99	11.5	1.13	184
9	CHO	**1c**	300	27	>99	9.7	1.31	127
10	CHO	**1d**	300	19	>99	11.4	1.22	138
11	CHO	**1e**	300	19	>99	11.3	1.17	168
12	CHO	**1f**	300	19	>99	11.6	1.23	156

[a]Reactions were performed in the presence of 1.3 mmol anhydride and excess epoxide: 0.55 mL neat BO (6.4 mmol) for entry 1-6; 0.39 mL CHO (3.9 mmol) and 0.2 mL toluene for entry 7-12. For all entries, [Al cat]:[PPNCl] = 1:0.9. [b]Conversion of anhydride determined by ^{1}H NMR spectroscopy. [c]Determined by GPC in THF at 30 °C, calibrated with polystyrene standards. [d]Determined by DSC; reported T_g values are from the second heat.

Supplementary Table 3 | Average molecular hole volumes ($<V_h>$) of **1a**–**1f**/PO copolymers.

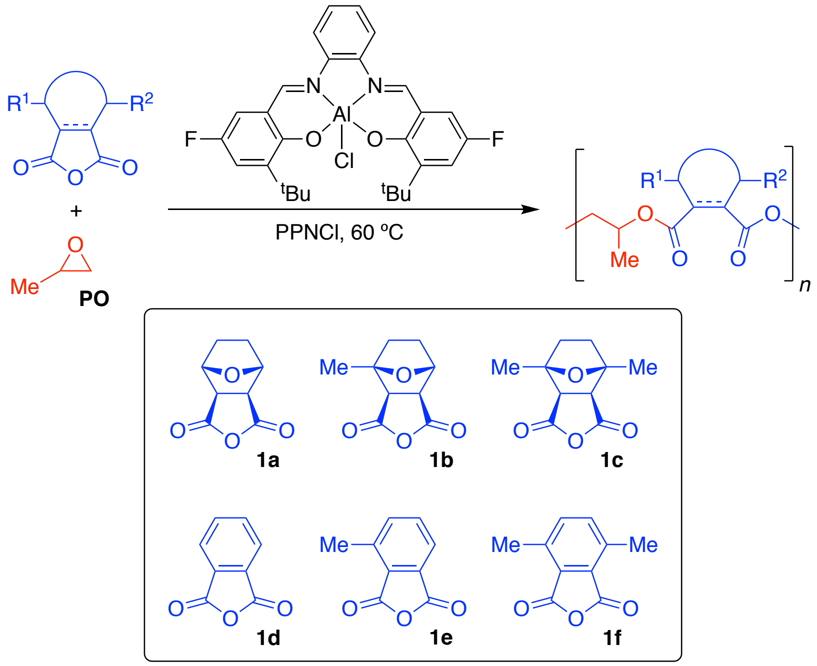

**sample**[a]	M_n **(kDa)**[b]	M_w/M_n[b]	T_g **(°C)**[c]	$<V_h>$ **(Å³)**[b]
Poly (**1a**-*alt*-PO)	23.0	1.21	100	67
Poly (**1b**-*alt*-PO)	32.0	1.22	120	119
Poly (**1c**-*alt*-PO)	28.9	1.24	93	99
Poly (**1d**-*alt*-PO)	20.0	1.21	63	71
Poly (**1e**-*alt*-PO)	19.0	1.09	91	84
Poly (**1f**-*alt*-PO)	17.0	1.07	85	78

[a]Polymer samples are from Table S1, entry 4-9, respectively to their structure. [b]Determined by GPC in THF at 30 °C, calibrated with polystyrene standards. [c]Determined by DSC; reported T_g values are from the second heat. [d]Determined by positron annihilation lifetime spectroscopy (PALS) under ambient conditions (temperature = 23 °C, relative humidity = 45%). Reported data are average values of seven independent measurements.

Supplementary Table 4 | Computed energy gaps between the global minimum and the most accessible TS structure in **2b** at several different levels of theory. All structures were optimized with B3LYP+D3(op)/6-311++G(d,p) as described in the main text.

	**6-311++G(d,p)**	**cc-pVDZ**	**aug-cc-pVDZ**	**cc-pVTZ**
B3LYP+D3(op)	3.82	3.80	3.81	3.54
MP2	-	4.26	4.09	3.80
CCSD	-	4.53	4.29	4.02

Supplementary Table 5 | Computed energy gaps between the global minimum and the most accessible TS structure in **2e** at several different levels of theory. All structures were optimized with B3LYP+D3(op)/6-311++G(d,p) as described in the main text.

	**6-311 ++G(d,p)**	**cc-pVDZ**	**aug-cc-pVDZ**	**cc-pVTZ**
B3LYP+D3(op)	3.73	3.87	3.92	3.93
MP2	-	4.12	3.98	4.17
CCSD	-	3.87	3.77	3.96

Supplementary Table 6 | Value of the θ and φ dihedral angles (in °) corresponding to the global minimum conformations of **2a-2f**.

**Structure**	$\boldsymbol{\theta}$	$\boldsymbol{\varphi}$
**2a**	227.2	233.4
**2b**	222.1	233.1
**2c**	218.5	232.7
**2d**	216.2	219.5
**2e**	102.9	356.5
**2f**	225.5	228.5

Supplementary Table 7 | Intramolecular SAPT interaction energies (kcal/mol) corresponding to steric interactions between the vicinal ester groups (E_M), the ester and methyl (if present) groups

on the left (E_L), and the ester and methyl (if present) groups on the right (E_R), as well as the relative ring strain energy (E_S). For each conformation, the total strain energy from all of these interactions is denoted by $E_{tot} = E_M + E_L + E_R + E_S$. For each structure, ΔE represents the change of total strain energy upon methyl substitution for the corresponding minimum (MIN) and each of its neighboring transition state (TSx) conformations (Fig. 5). Red and green entries represent the highest and lowest ΔE values, respectively, among the conformations corresponding to a given structure.

Structure	Conformation	E_M	E_L	E_R	E_S	E_{tot}	ΔE
**2a**	MIN	**7.49**	-	-	**1.72**	9.21	-
	TS1	8.41	-	-	2.40	10.81	-
	TS2	10.59	-	-	1.95	12.54	-
	TS3	8.61	-	-	2.26	10.87	-
**2b**	MIN	**7.61**	5.14	-	**3.28**	16.03	6.82
	TS1	8.46	7.71	-	3.66	19.83	**9.02**
	TS2	10.48	5.05	-	3.95	19.48	**6.94**
	TS3	9.23	5.69	-	3.85	18.77	**7.90**
**2c**	MIN	**7.57**	5.34	7.32	**5.09**	25.32	**9.29**
	TS1	9.11	7.00	5.44	6.11	27.66	7.83
	TS2	11.28	4.90	5.69	5.04	26.91	7.43
	TS3	8.89	5.18	6.58	6.21	26.86	8.09
**2d**	MIN	**6.69**	-	-	**0.21**	6.90	-
	TS1	7.52	-	-	0.24	7.76	-
	TS2	7.52	-	-	0.24	7.76	-
**2e**	MIN	**7.33**	6.34	-	**0.44**	14.11	7.21
	TS1	8.13	8.06	-	0.70	16.89	**9.13**
	TS2	8.65	7.42	-	0.73	16.80	**9.04**
**2f**	MIN	**7.85**	6.66	6.50	**0.82**	21.83	**7.72**
	TS1	8.83	8.19	5.87	0.99	23.88	6.99
	TS2	8.83	5.87	8.19	0.99	23.88	7.08

Supplementary Figures

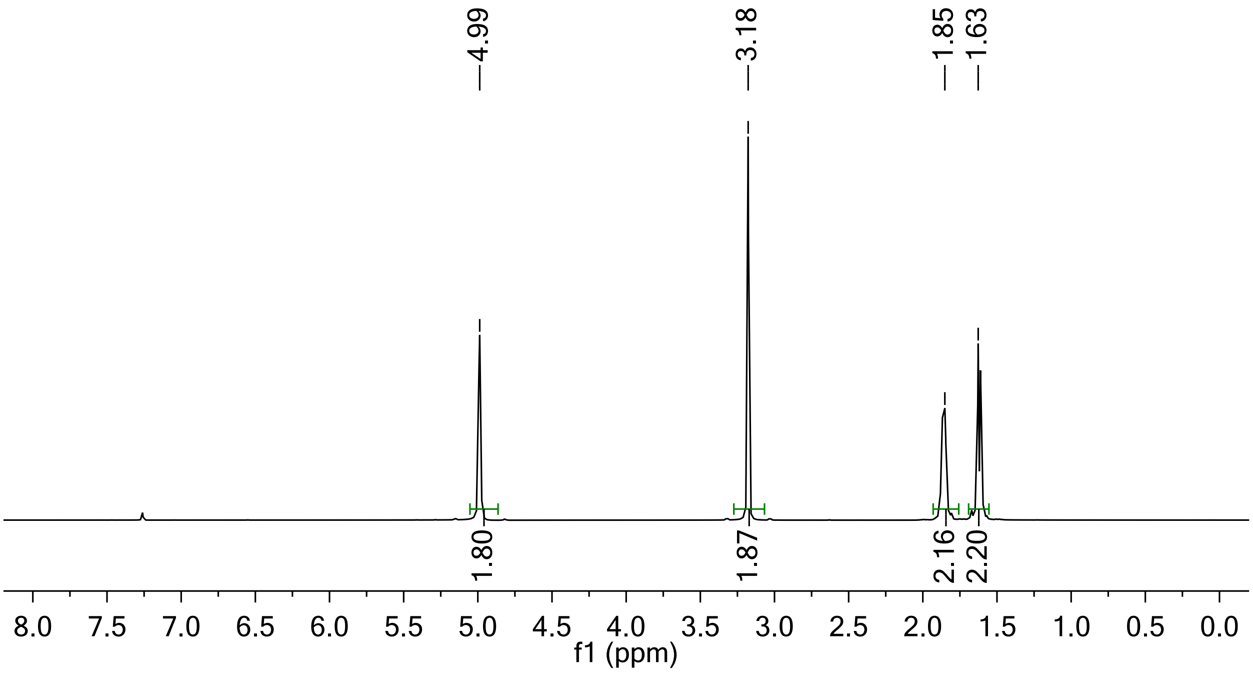

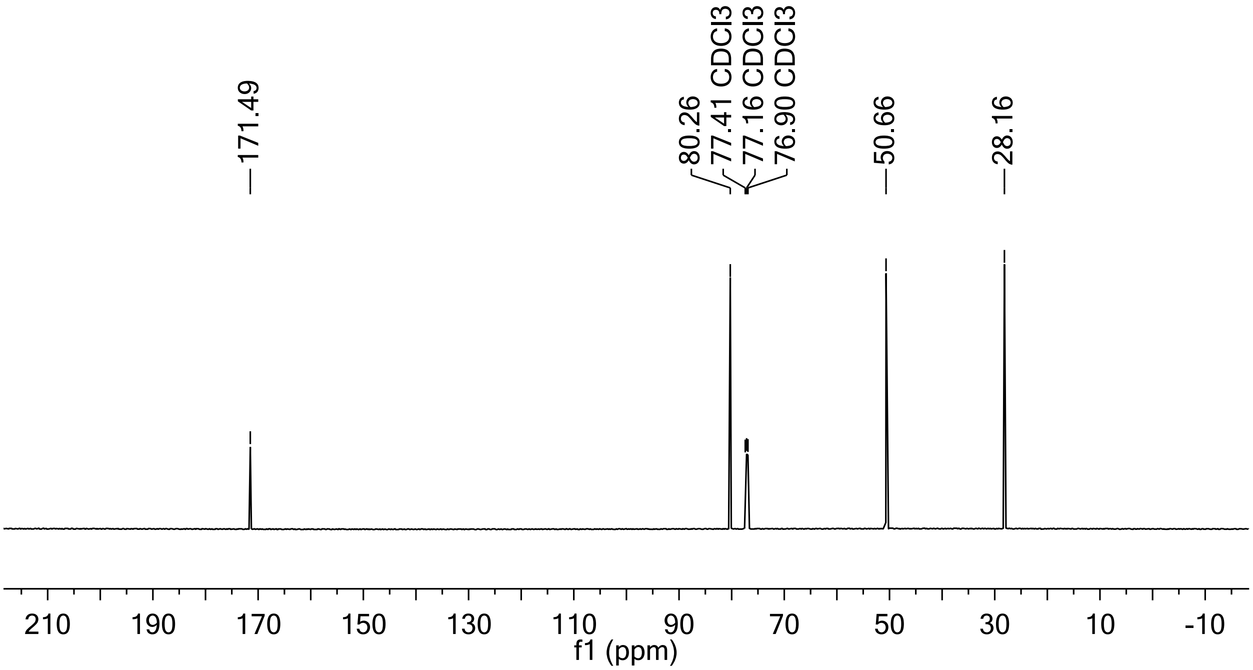

Supplementary Figure 1 | ^{1}H NMR (top) and ^{13}C NMR (bottom) spectrum for *cis*-*exo*-7-oxabicyclo[2.2.1]heptane-2,3-dicarboxylic anhydride (**1a**).

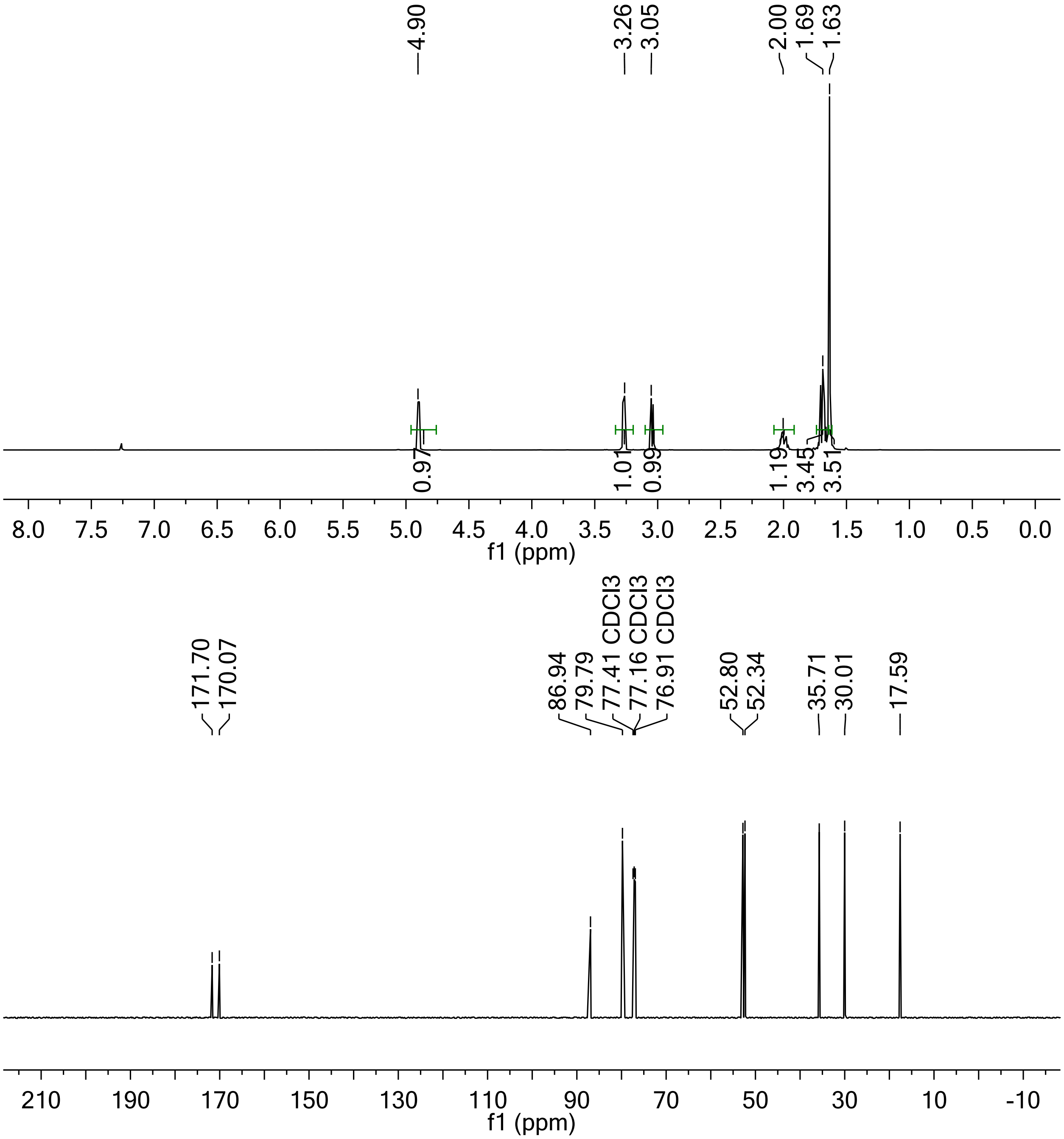

Supplementary Figure 2 | ^{1}H NMR (top) and ^{13}C NMR (bottom) spectrum for *cis*-*exo*-1-methyl-7-oxabicyclo[2.2.1]heptane-2,3-dicarboxylic anhydride (**1b**).

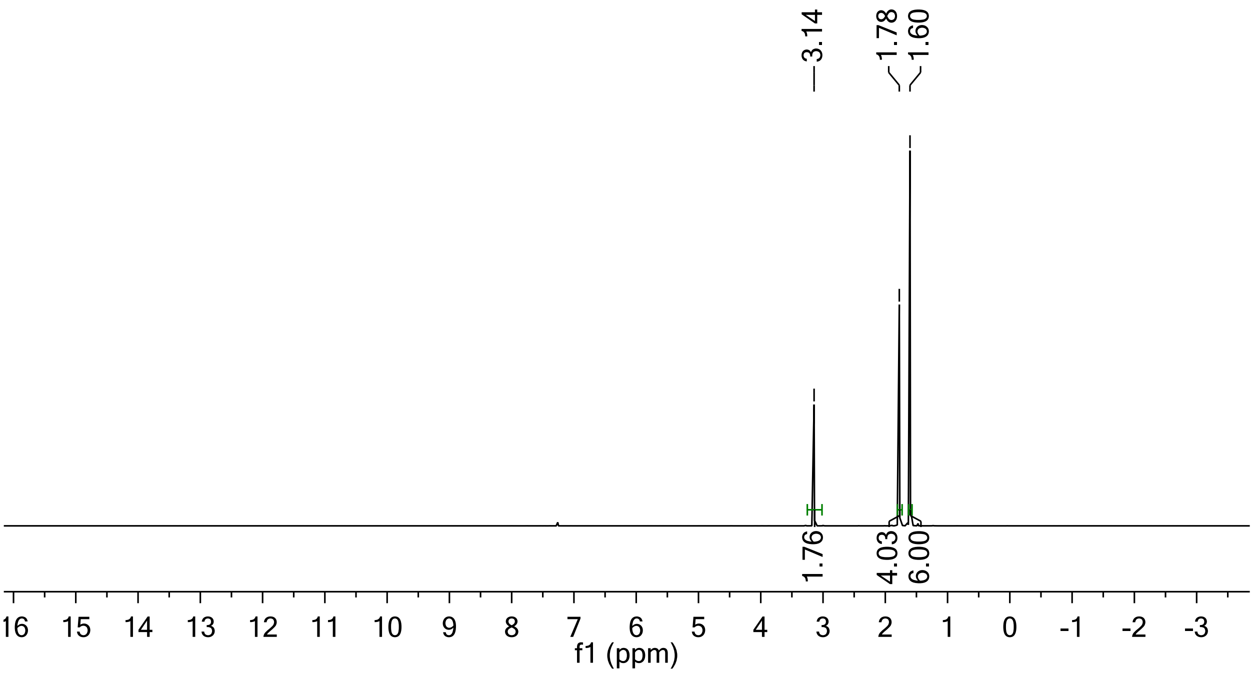

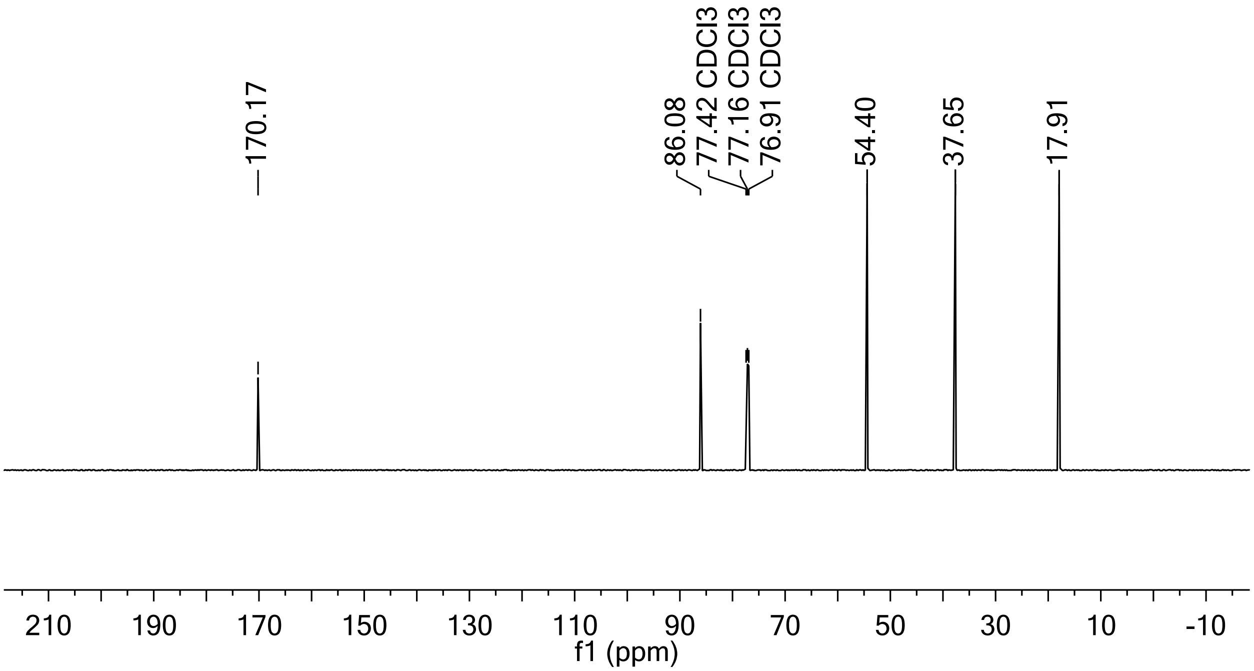

Supplementary Figure 3 | ^{1}H NMR (top) and ^{13}C NMR (bottom) spectrum for *cis*-*exo*-1,4-dimethyl-7-oxabicyclo[2.2.1]heptane-2,3-dicarboxylic anhydride (**1c**).

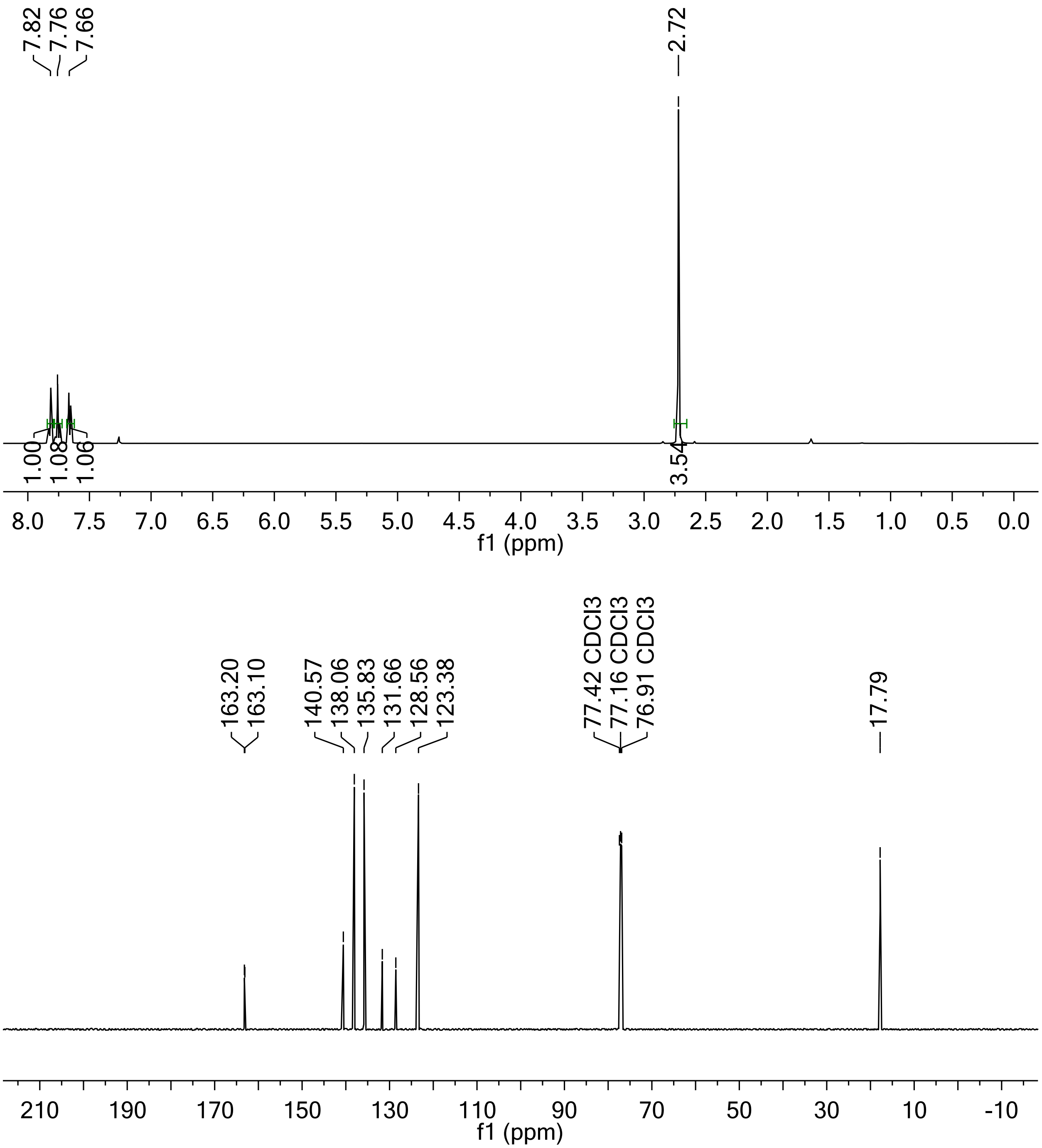

Supplementary Figure 4 | ^{1}H NMR (top) and ^{13}C NMR (bottom) spectrum for 3-methylphthalic anhydride (**1e**).

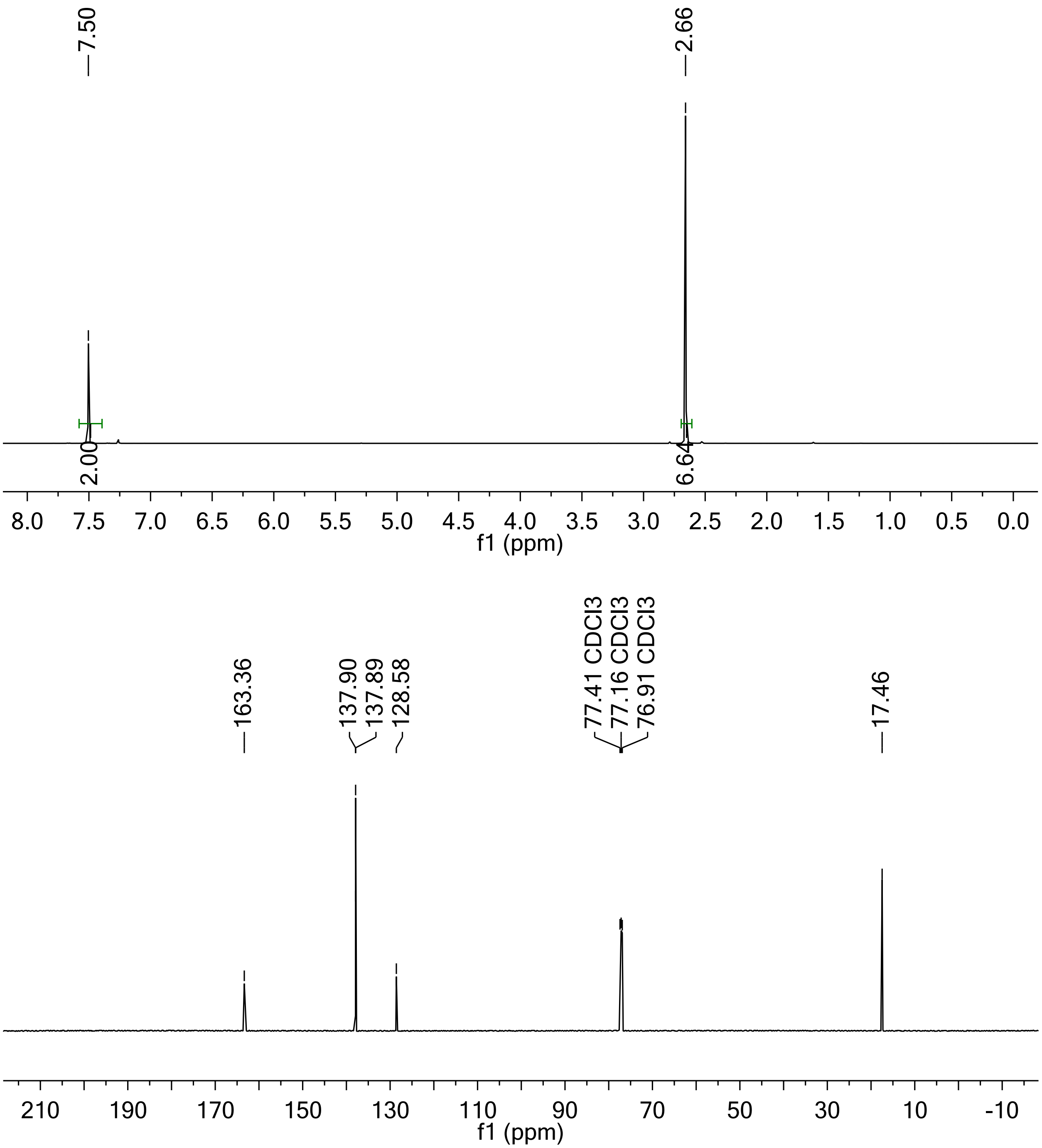

Supplementary Figure 5 | ^{1}H NMR (top) and ^{13}C NMR (bottom) spectrum for 3,6-dimethylphthalic anhydride (**1f**).

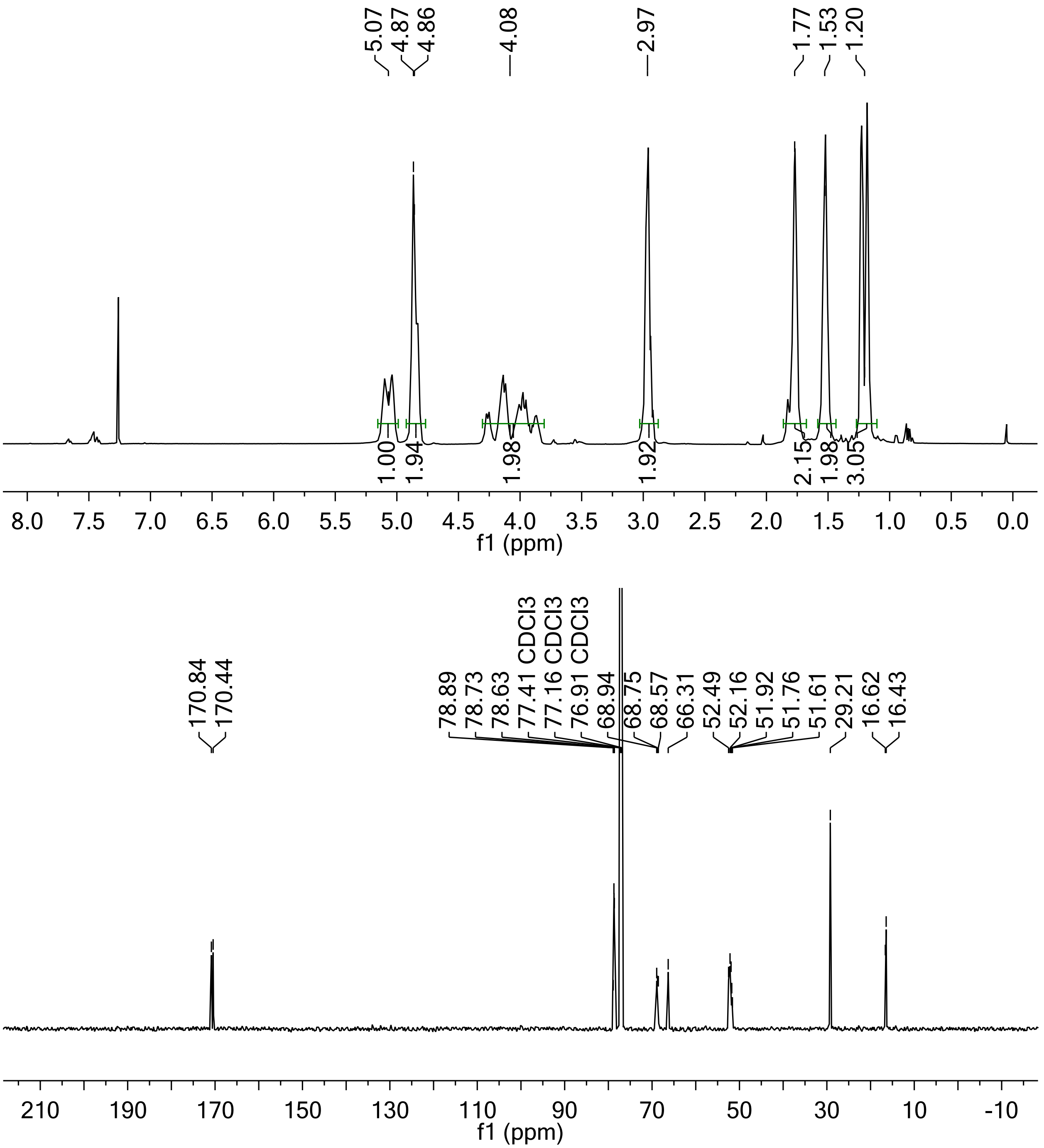

Supplementary Figure 6 | ^{1}H NMR (top) and ^{13}C NMR (bottom) spectrum for poly(**1a**-*alt*-PO) (Supplementary Table 1, entry 4).

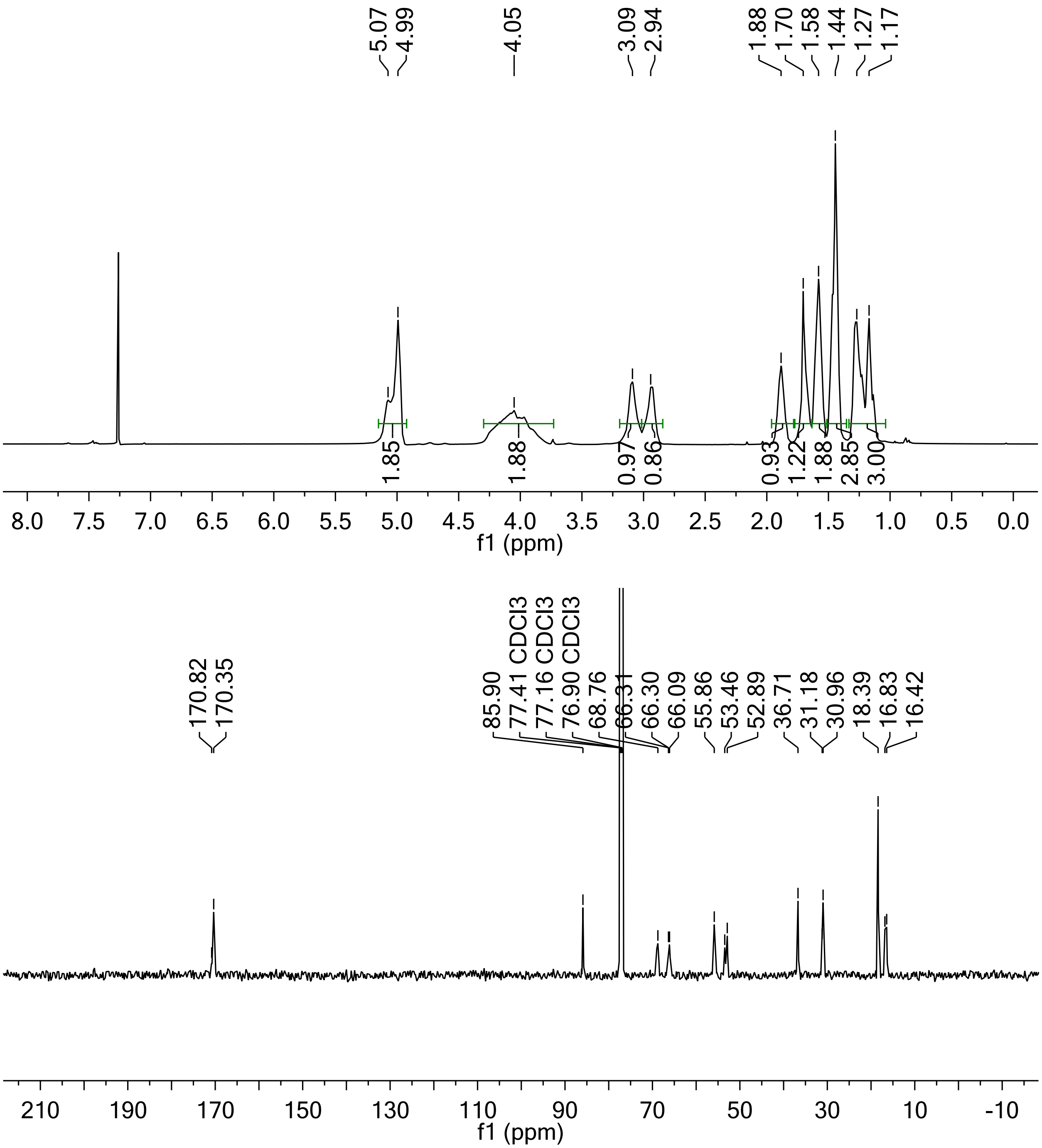

Supplementary Figure 7 | ^{1}H NMR (top) and ^{13}C NMR (bottom) spectrum for poly(**1b**-*alt*-PO) (Supplementary Table 1, entry 5).

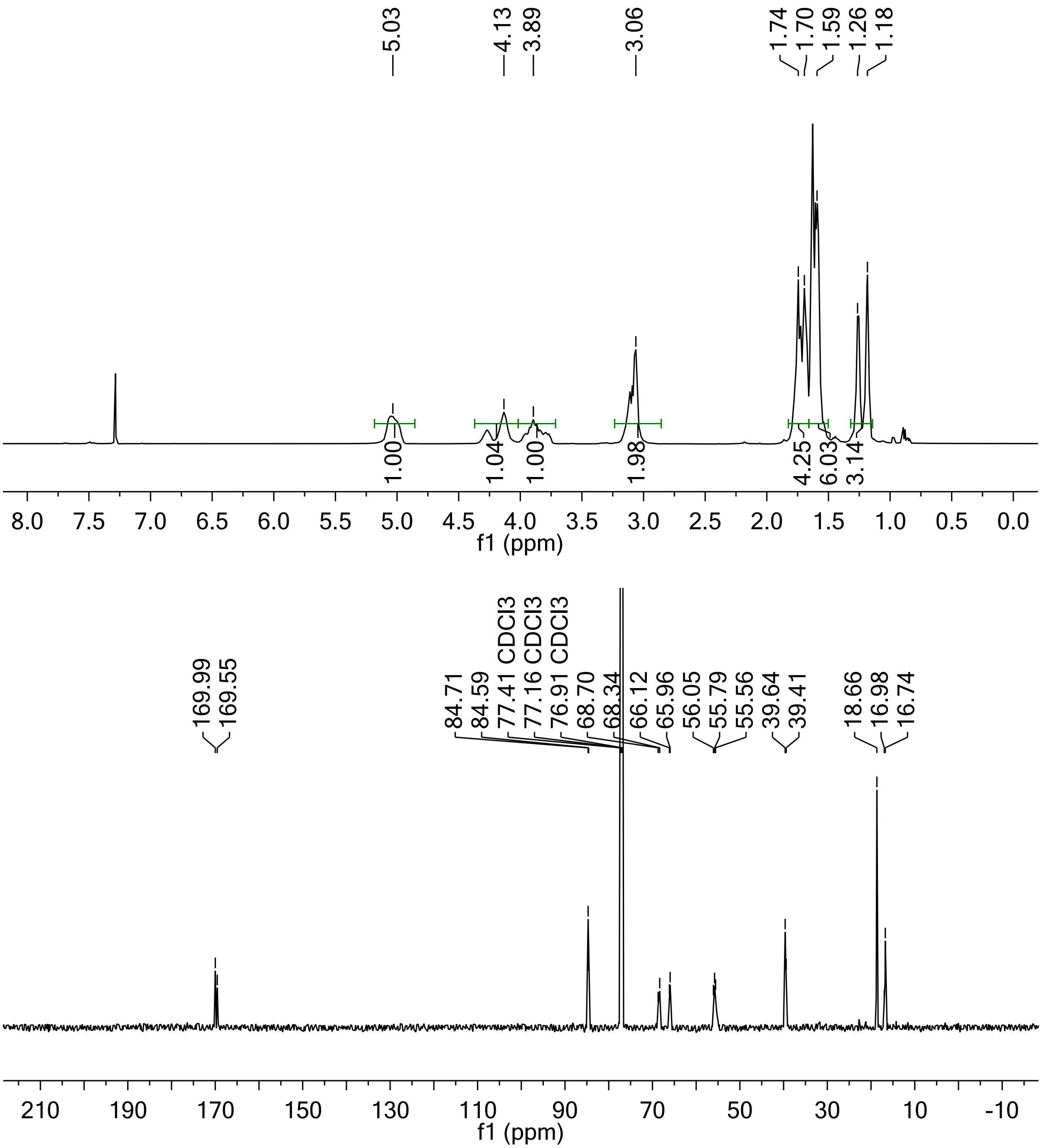

Supplementary Figure 8 | ^{1}H NMR (top) and ^{13}C NMR (bottom) spectrum for poly(**1c**-*alt*-PO) (Supplementary Table 1, entry 6).

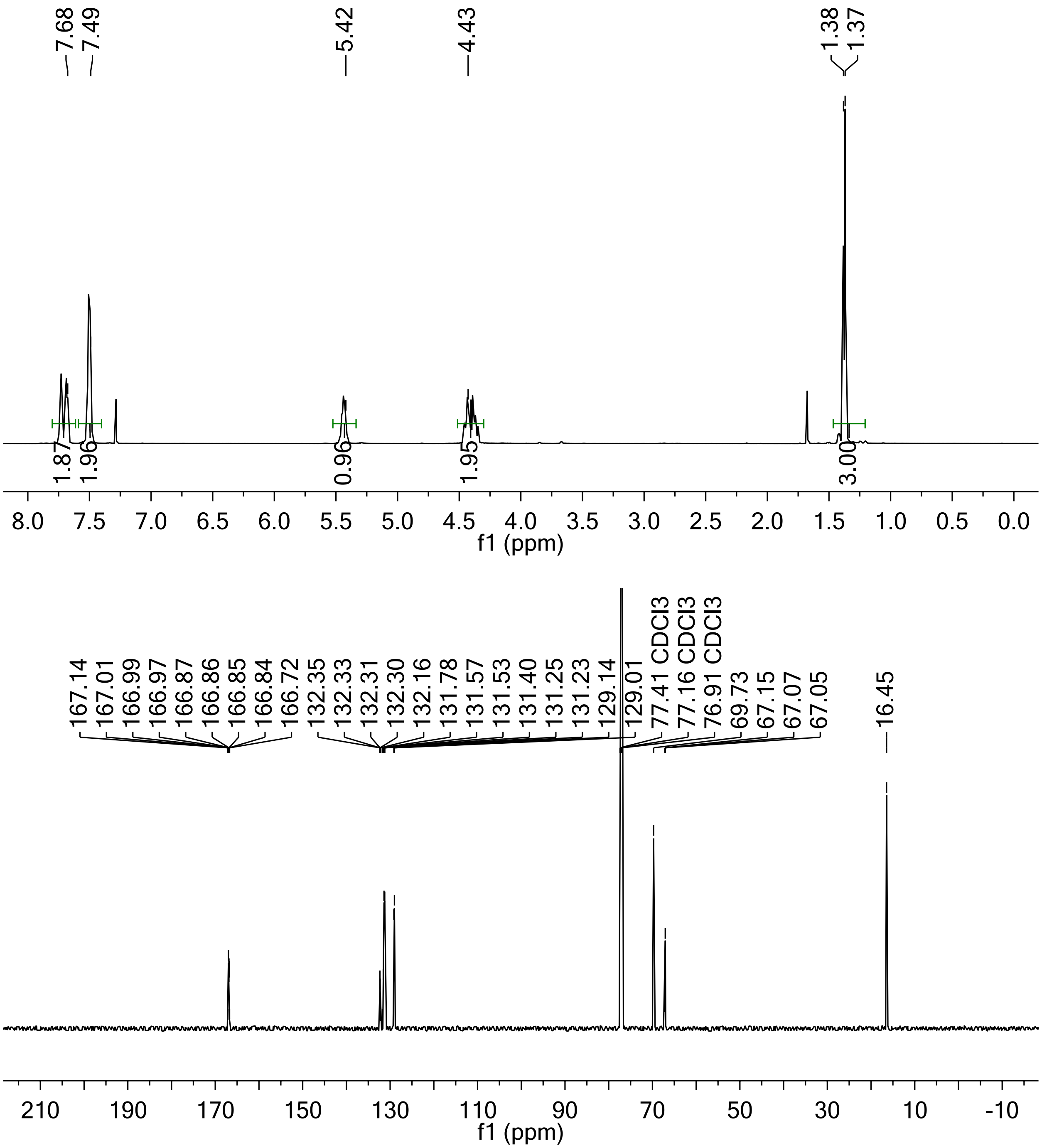

Supplementary Figure 9 | ^{1}H NMR (top) and ^{13}C NMR (bottom) spectrum for poly(**1d**-*alt*-PO) (Supplementary Table 1, entry 7).

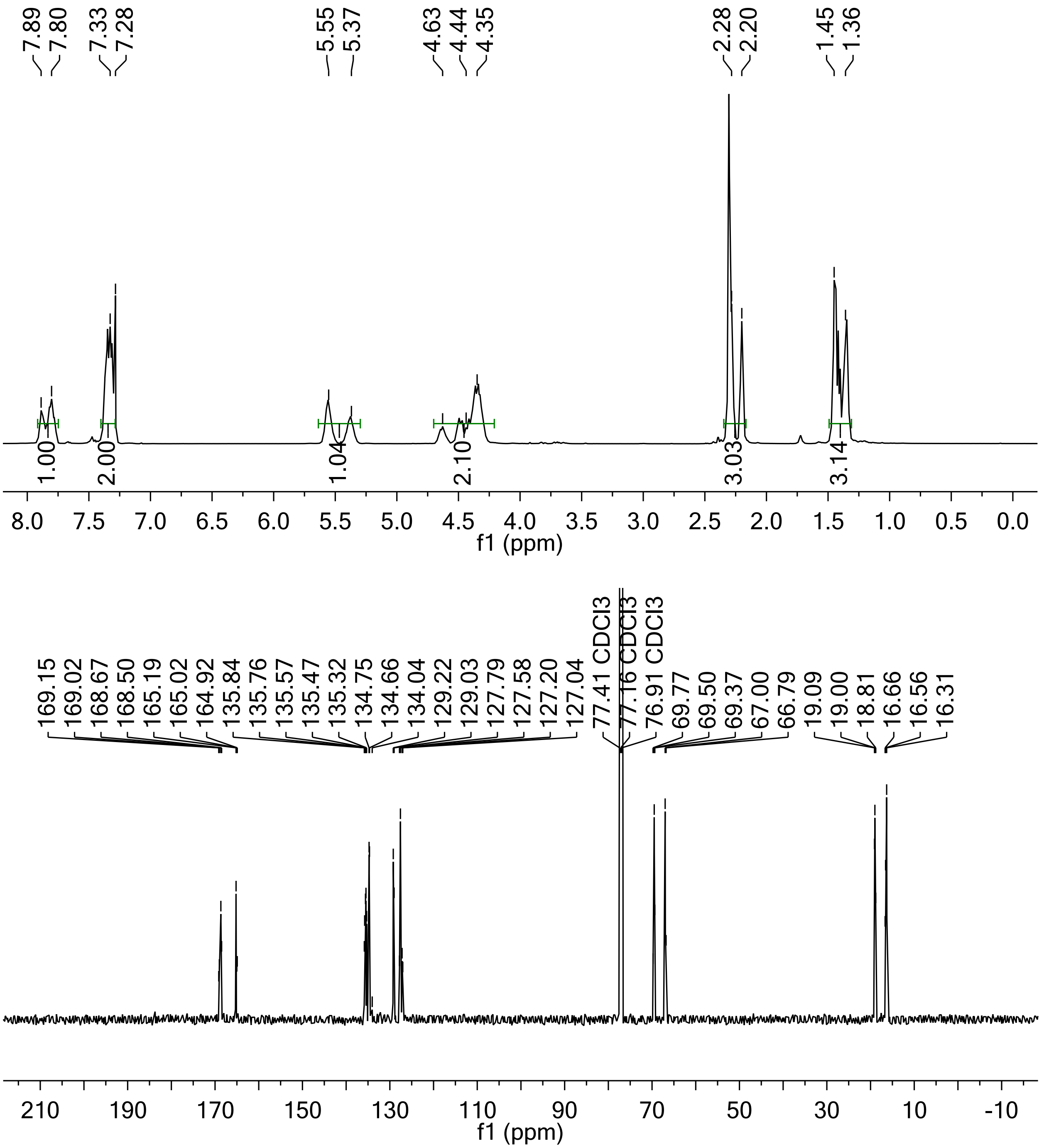

Supplementary Figure 10 | ^{1}H NMR (top) and ^{13}C NMR (bottom) spectrum for poly(**1e**-*alt*-PO) (Supplementary Table 1, entry 8).

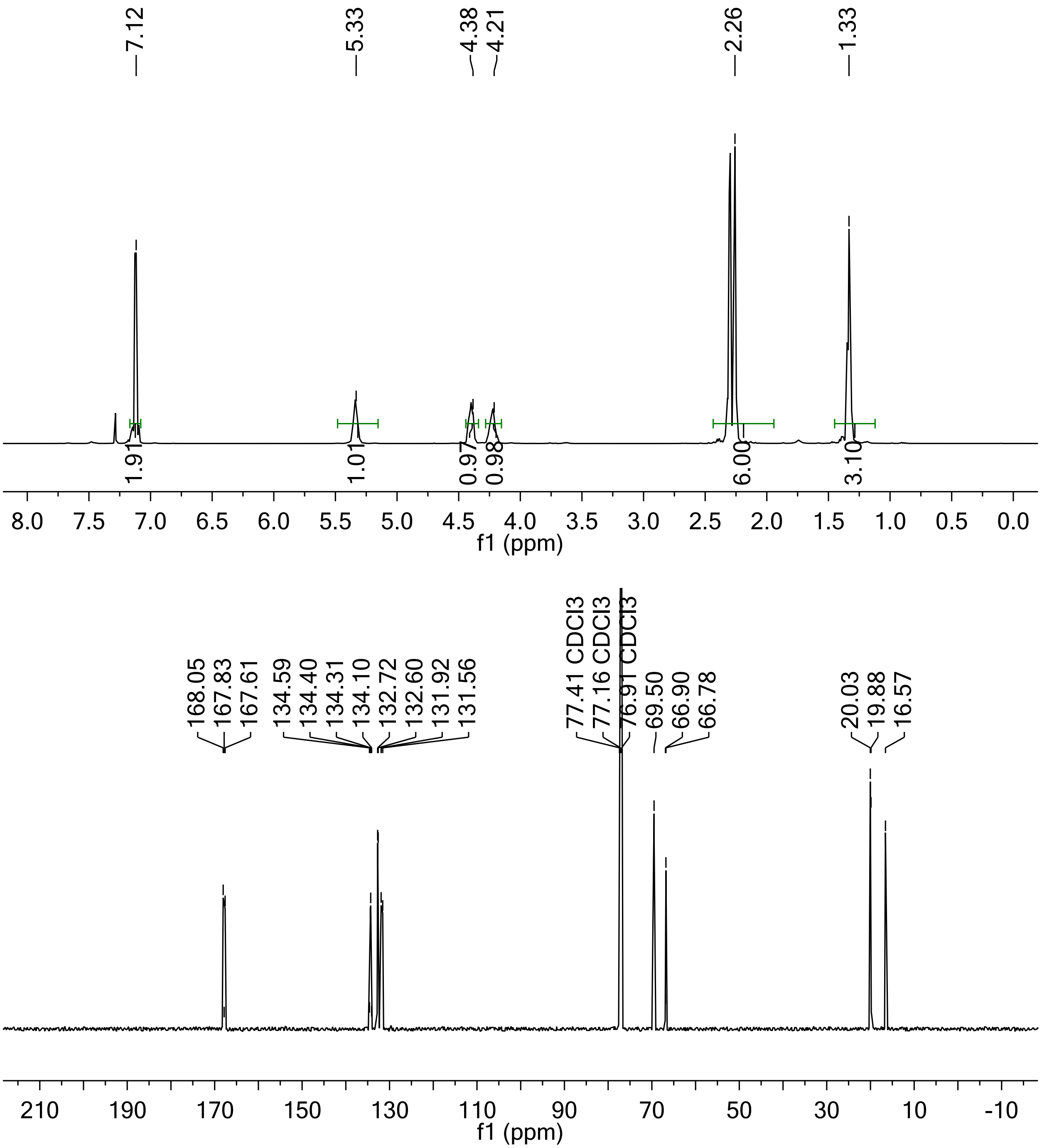

Supplementary Figure 11 | ^{1}H NMR (top) and ^{13}C NMR (bottom) spectrum for poly(**1f**-*alt*-PO) (Supplementary Table 1, entry 9).

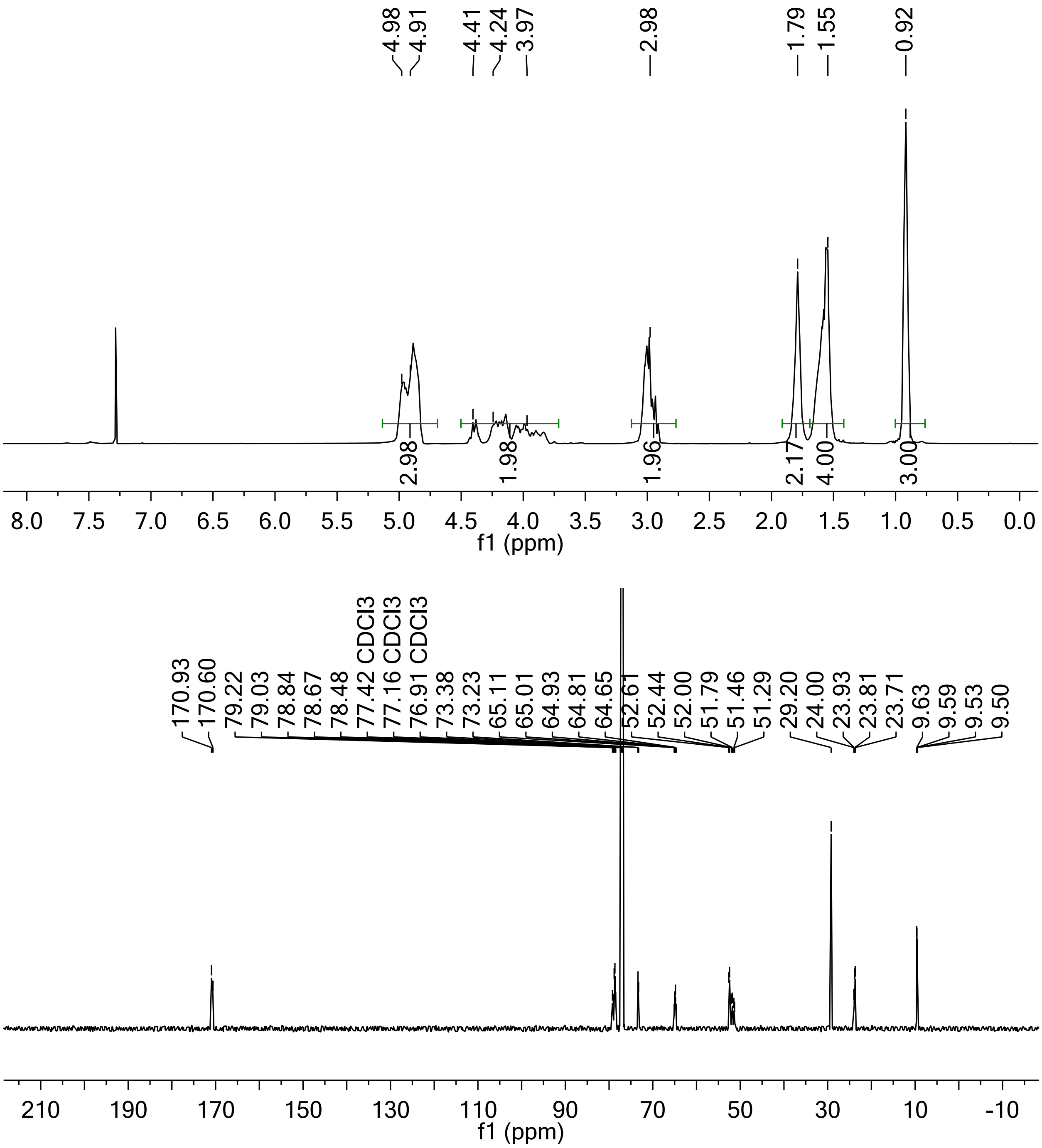

Supplementary Figure 12 | ^{1}H NMR (top) and ^{13}C NMR (bottom) spectrum for poly(**1a**-*alt*-BO) (Supplementary Table 2, entry 1).

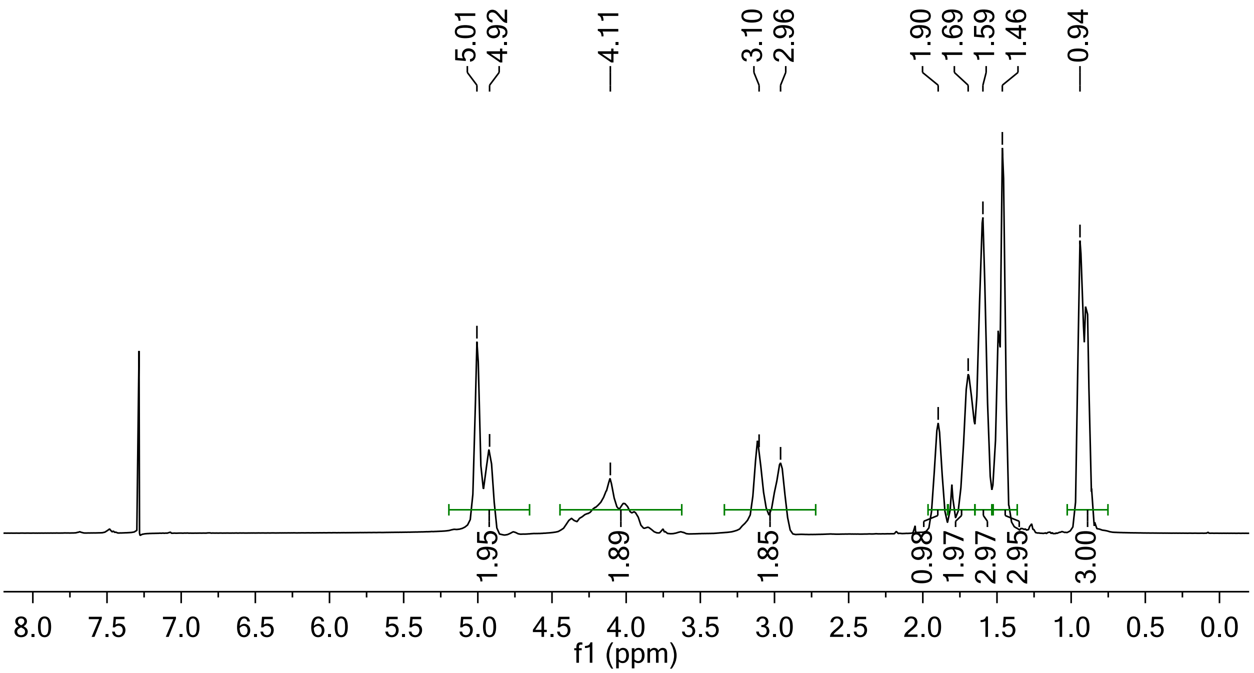

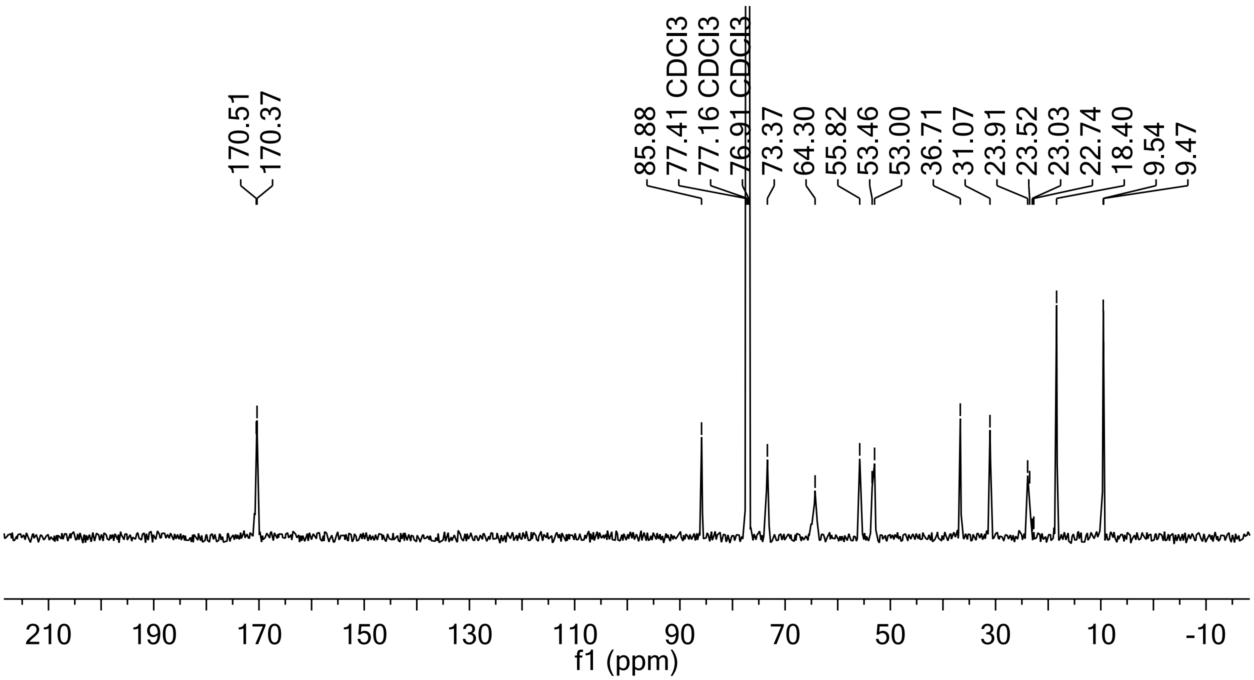

Supplementary Figure 13 | ^{1}H NMR (top) and ^{13}C NMR (bottom) spectrum for poly(**1b**-*alt*-BO) (Supplementary Table 2, entry 2).

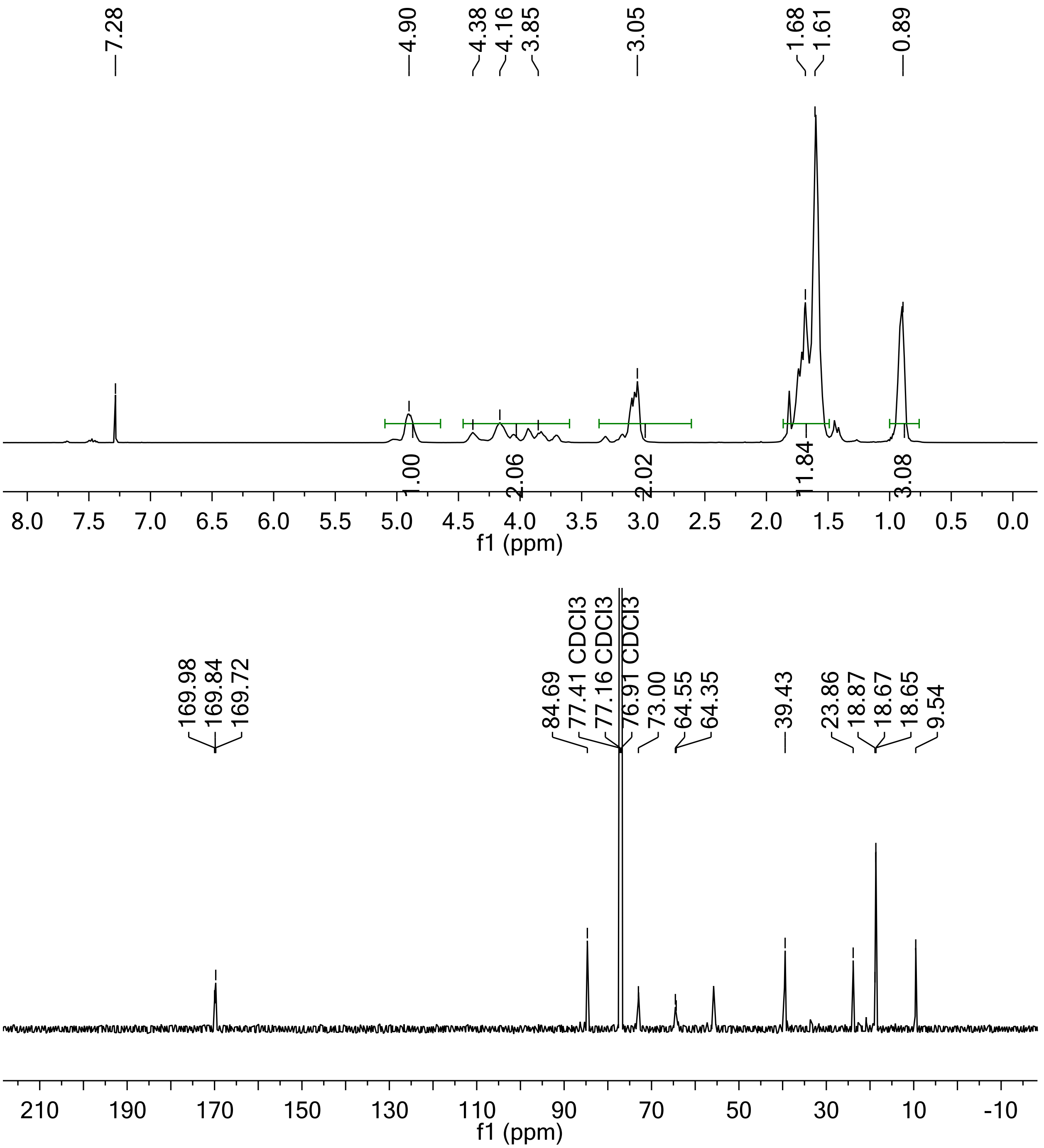

Supplementary Figure 14 | ^{1}H NMR (top) and ^{13}C NMR (bottom) spectrum for poly(**1c**-*alt*-BO) (Supplementary Table 2, entry 3).

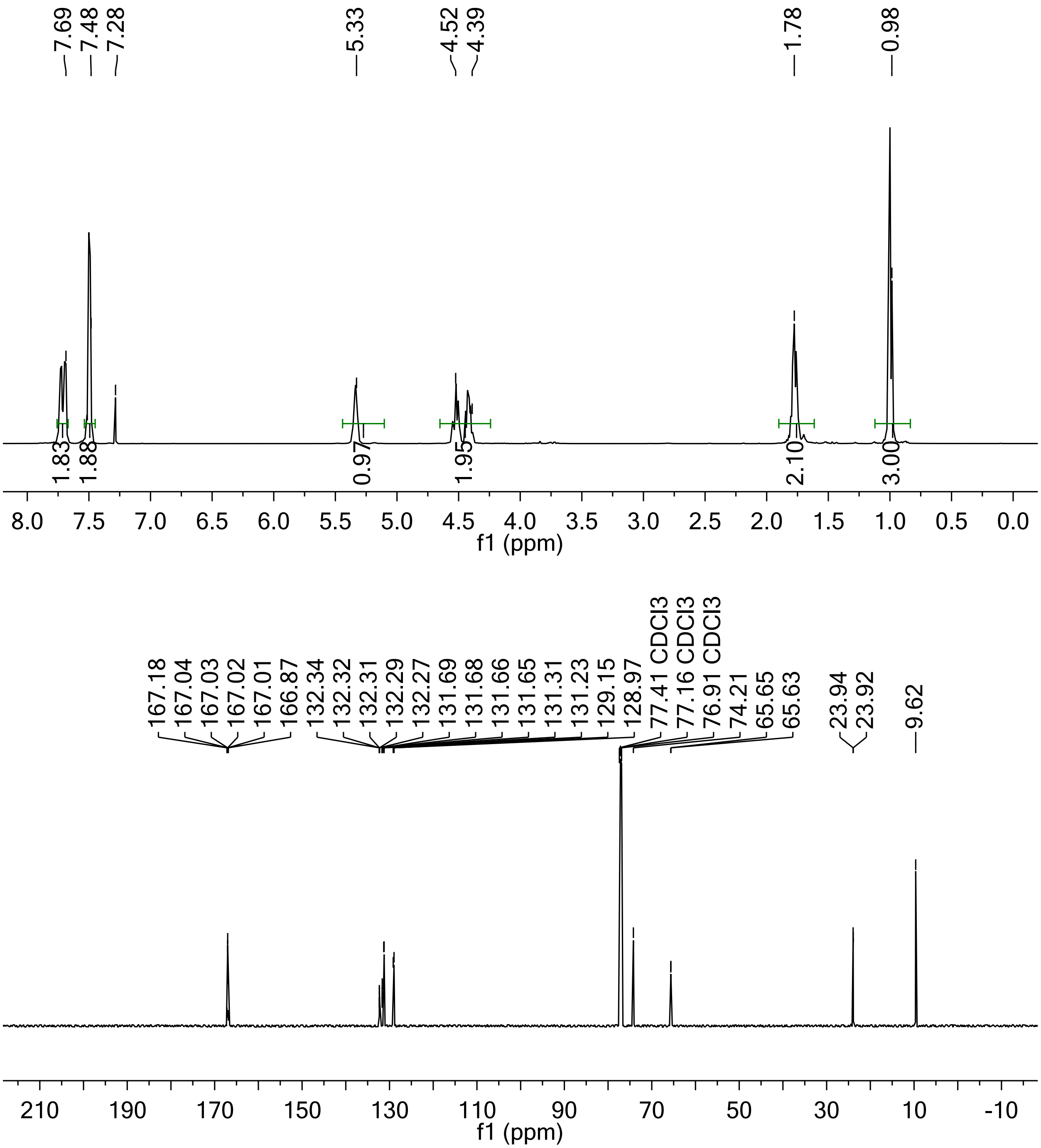

Supplementary Figure 15 | ^{1}H NMR (top) and ^{13}C NMR (bottom) spectrum for poly(**1d**-*alt*-BO) (Supplementary Table 2, entry 4).

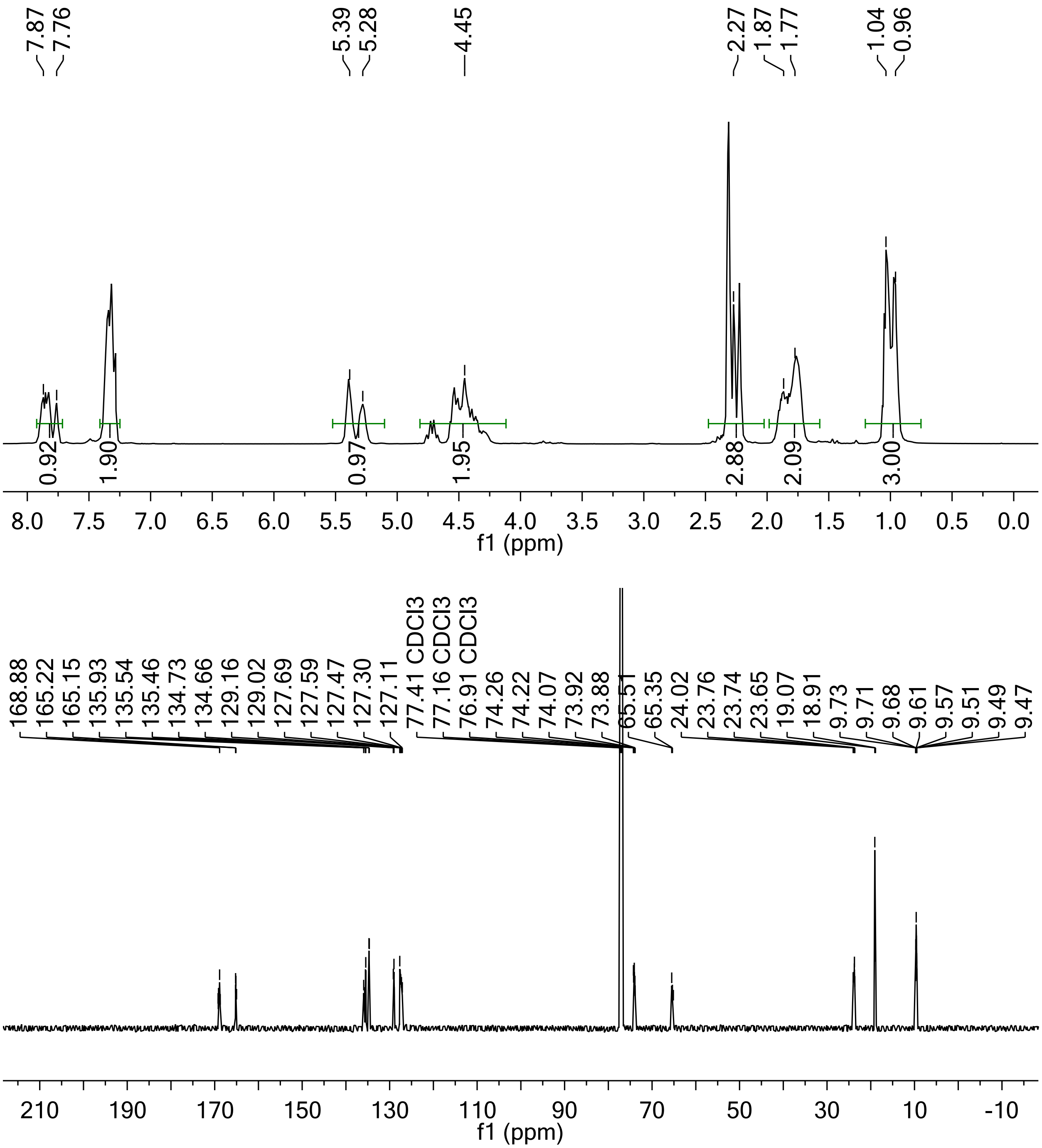

Supplementary Figure 16 | ^{1}H NMR (top) and ^{13}C NMR (bottom) spectrum for poly(**1e**-*alt*-BO) (Supplementary Table 2, entry 5).

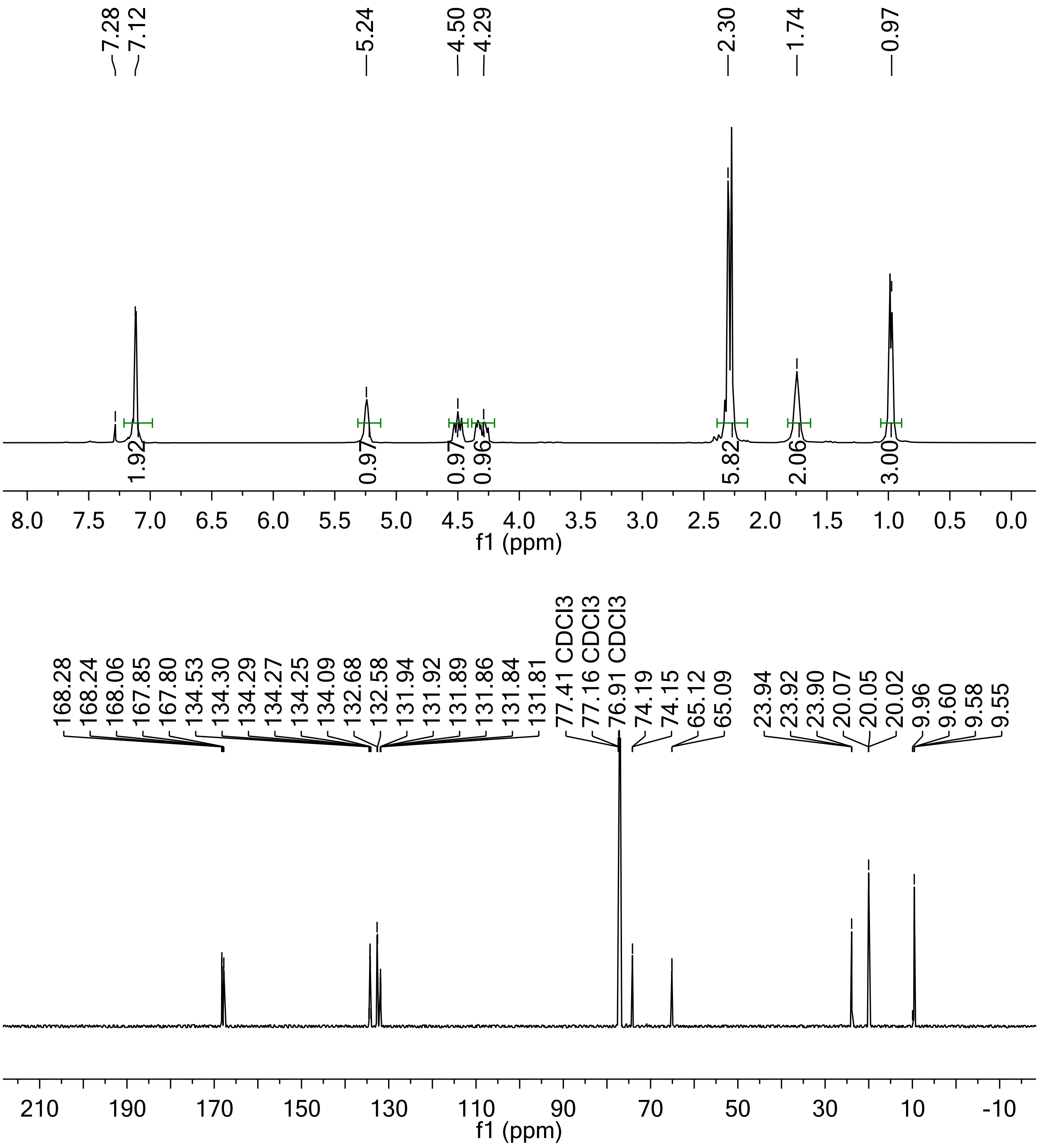

Supplementary Figure 17 | ^{1}H NMR (top) and ^{13}C NMR (bottom) spectrum for poly(**1f**-*alt*-BO) (Supplementary Table 2, entry 6).

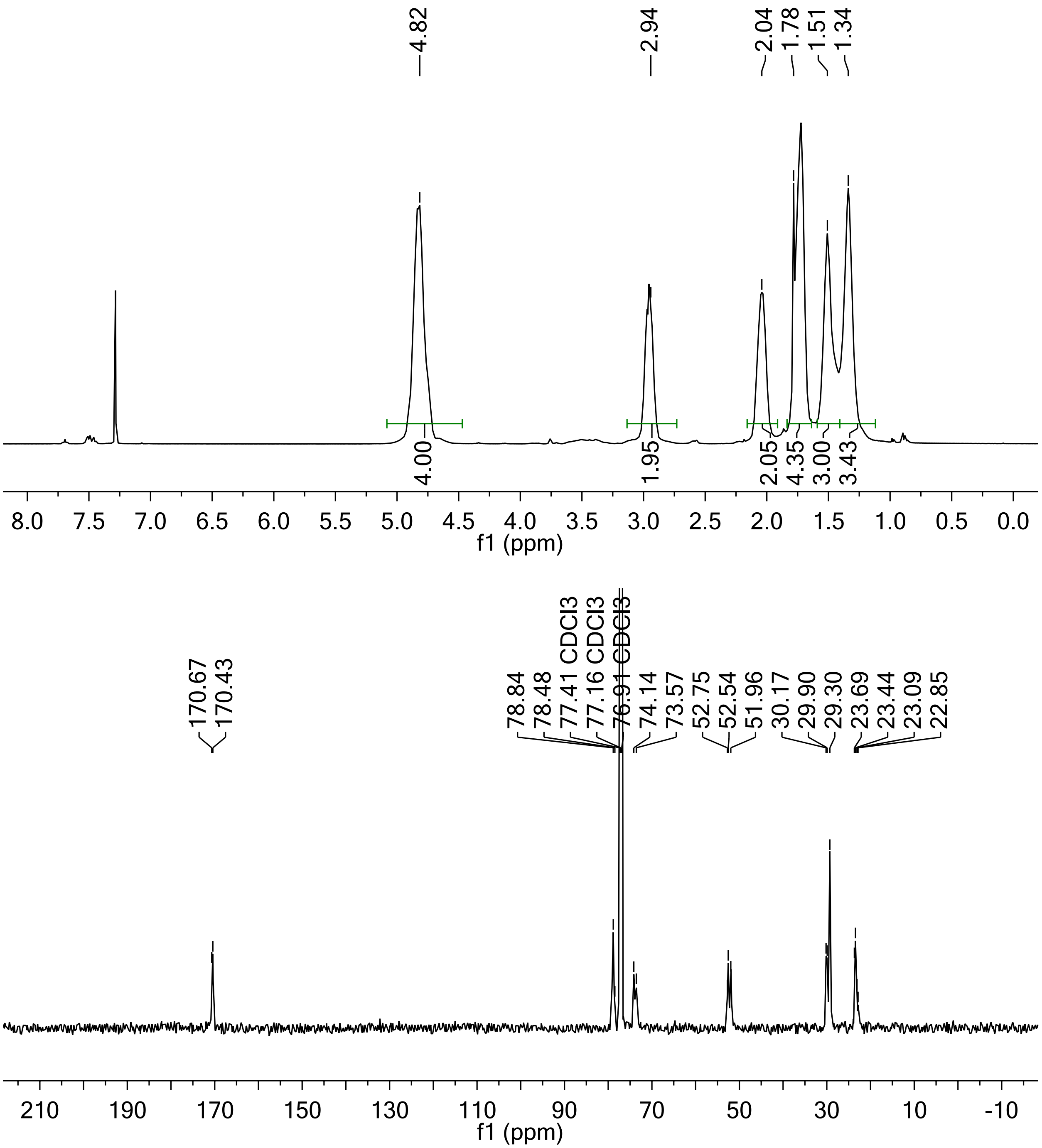

Supplementary Figure 18 | ^{1}H NMR (top) and ^{13}C NMR (bottom) spectrum for poly(**1a**-*alt*-CHO) (Supplementary Table 2, entry 7).

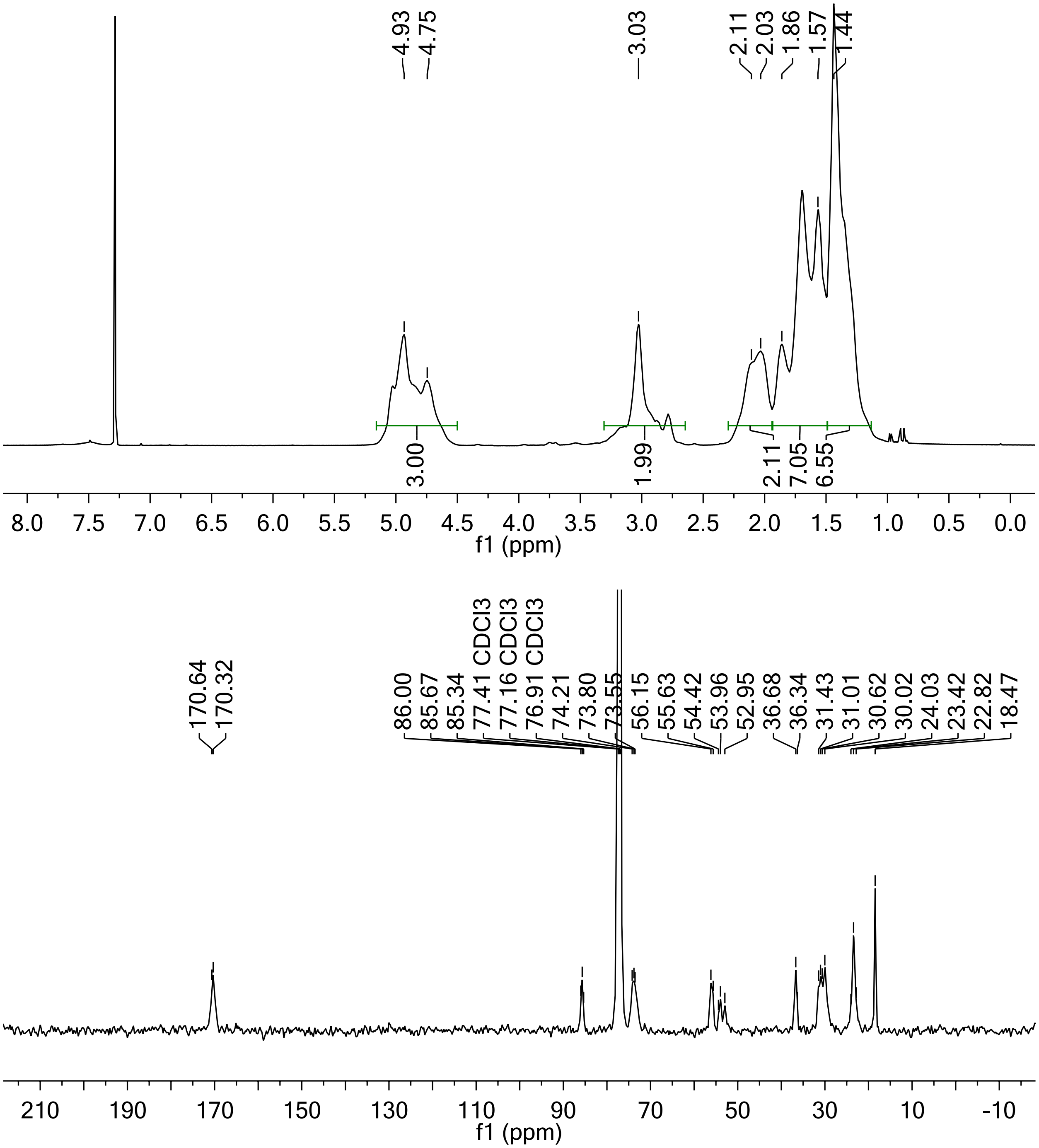

Supplementary Figure 19 | ^{1}H NMR (top) and ^{13}C NMR (bottom) spectrum for poly(**1b**-*alt*-CHO) (Supplementary Table 2, entry 8).

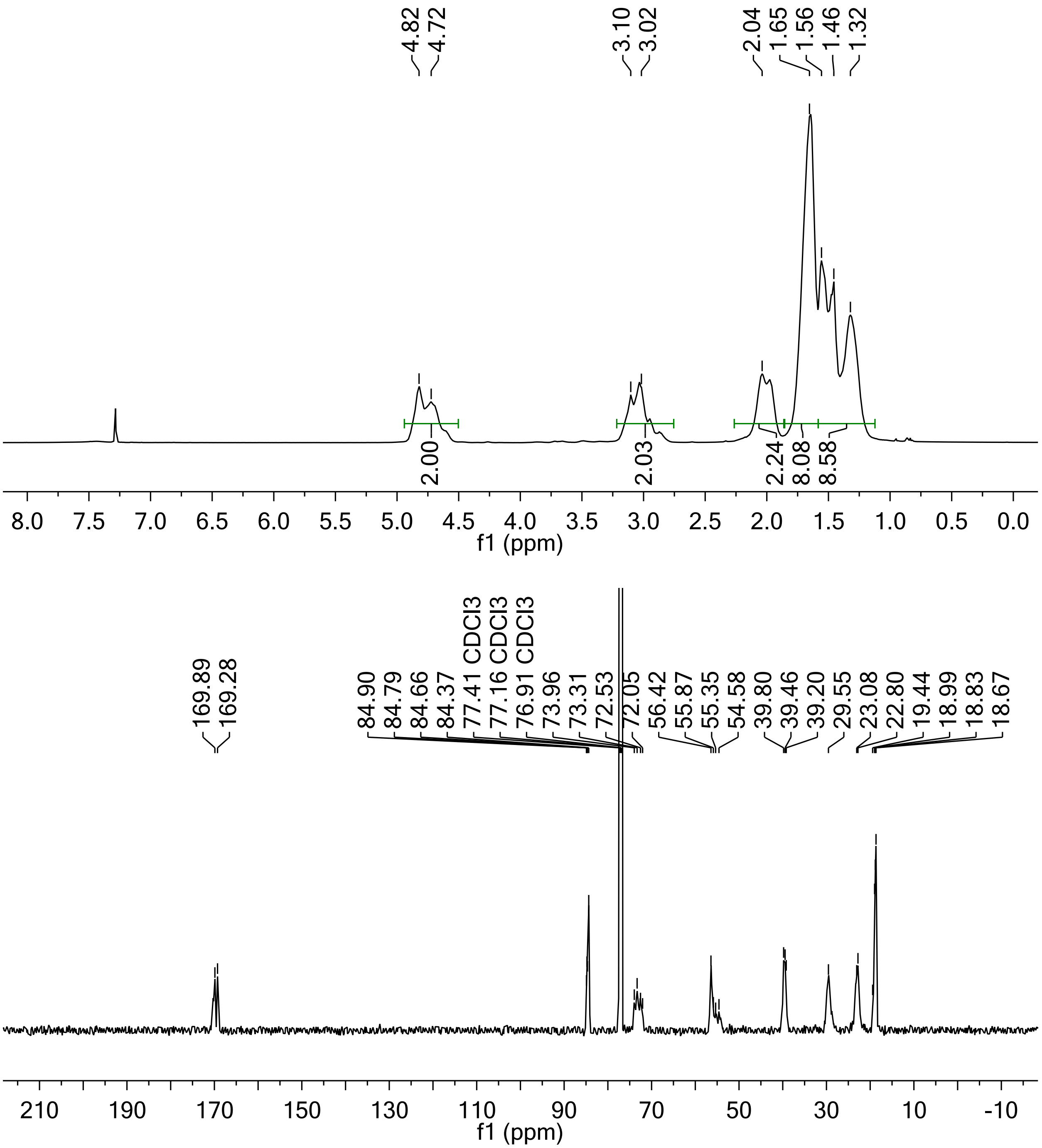

Supplementary Figure 20 | ^{1}H NMR (top) and ^{13}C NMR (bottom) spectrum for poly(**1c**-*alt*-CHO) (Supplementary Table 2, entry 9).

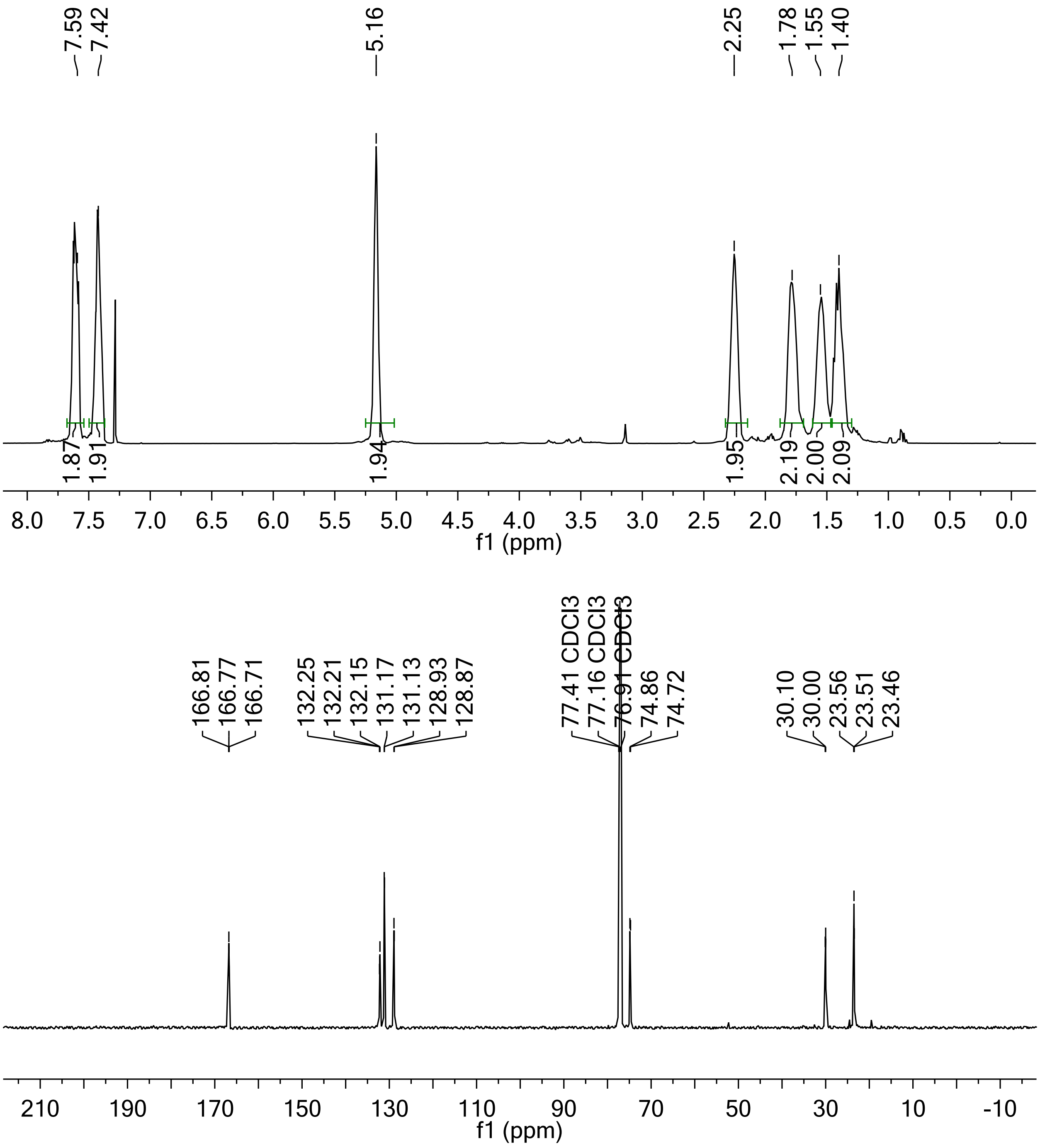

Supplementary Figure 21 | ^{1}H NMR (top) and ^{13}C NMR (bottom) spectrum for poly(**1d**-*alt*-CHO) (Supplementary Table 2, entry 10).

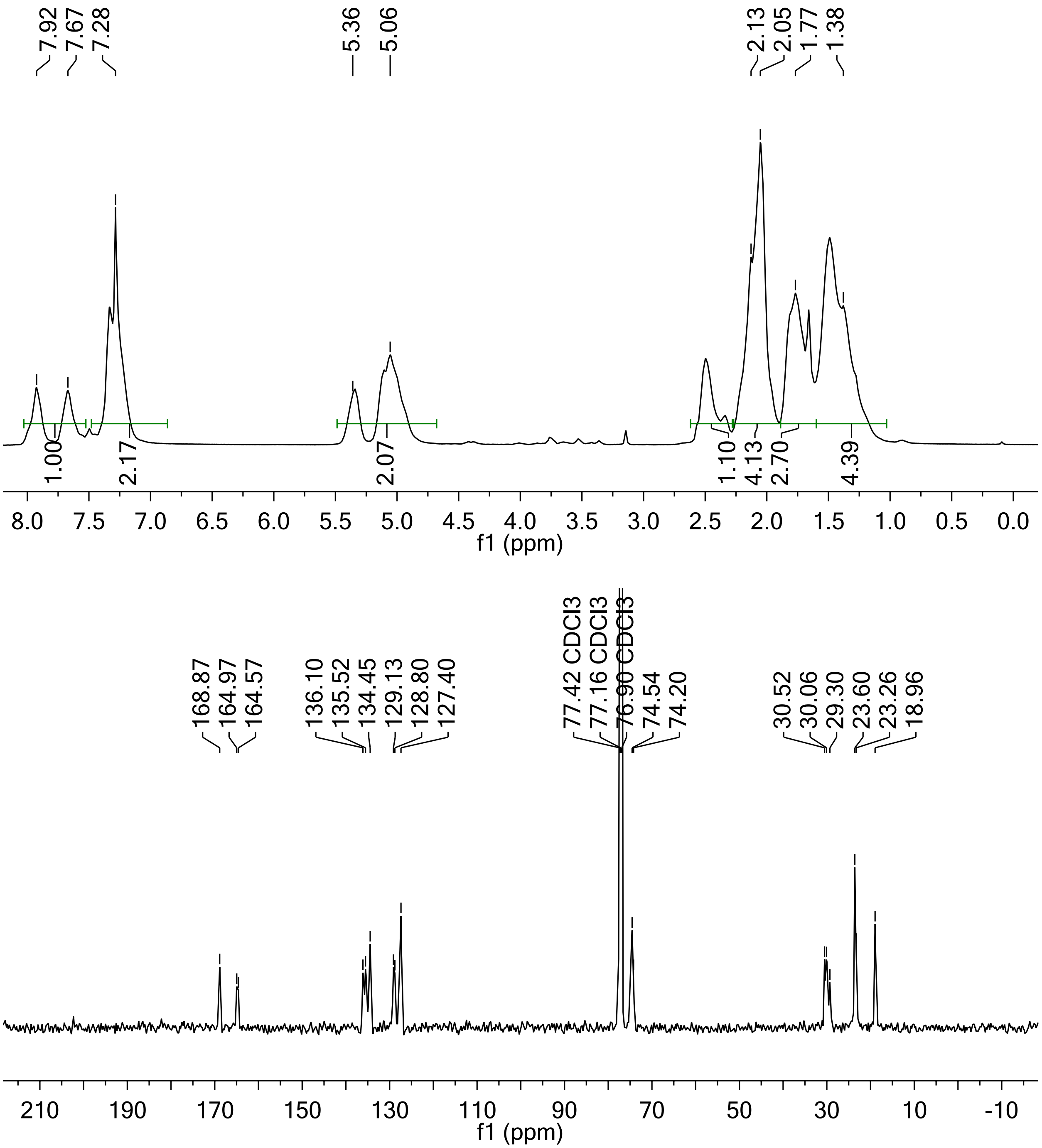

Supplementary Figure 22 | ^{1}H NMR (top) and ^{13}C NMR (bottom) spectrum for poly(**1e**-*alt*-CHO) (Supplementary Table 2, entry 11).

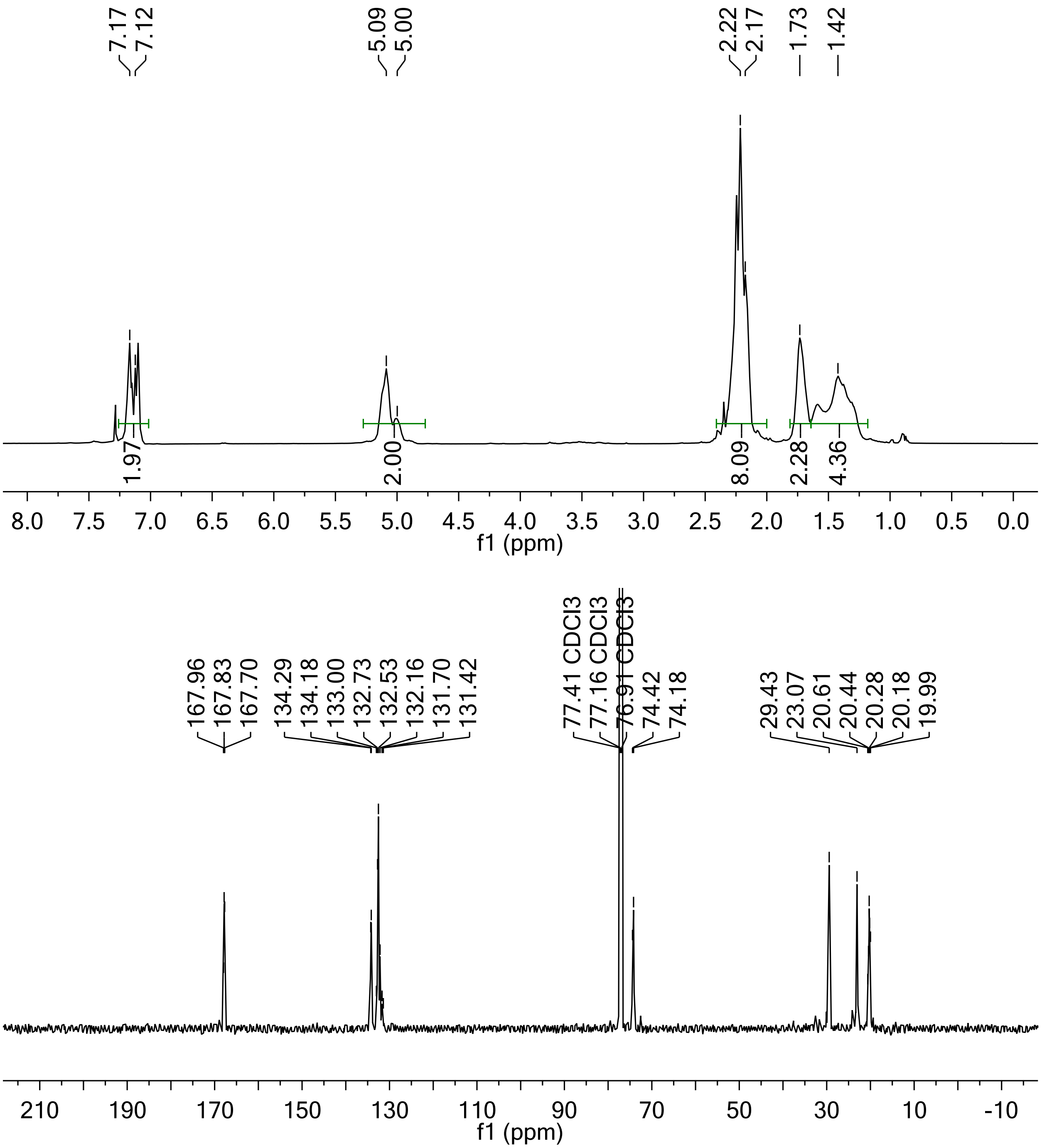

Supplementary Figure 23 | ^{1}H NMR (top) and ^{13}C NMR (bottom) spectrum for poly(**1f**-*alt*-CHO) (Supplementary Table 2, entry 12).

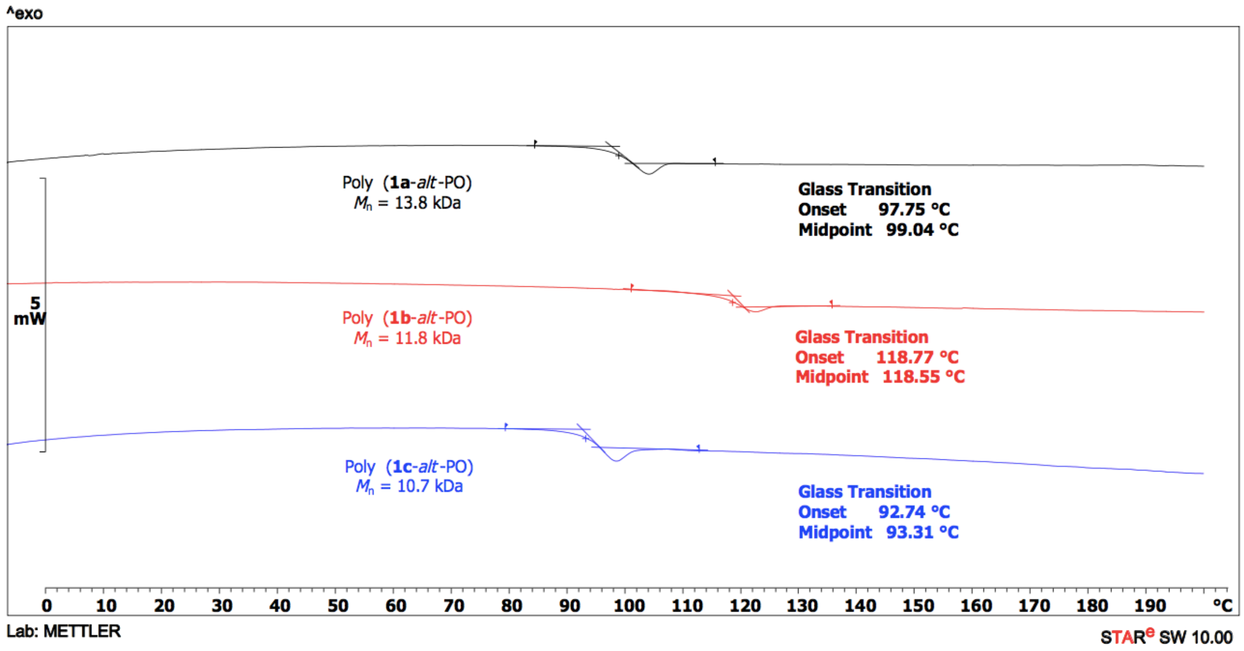

Supplementary Figure 24 | DSC thermograms of polymers in Supplementary Table 1, entry 1–3.

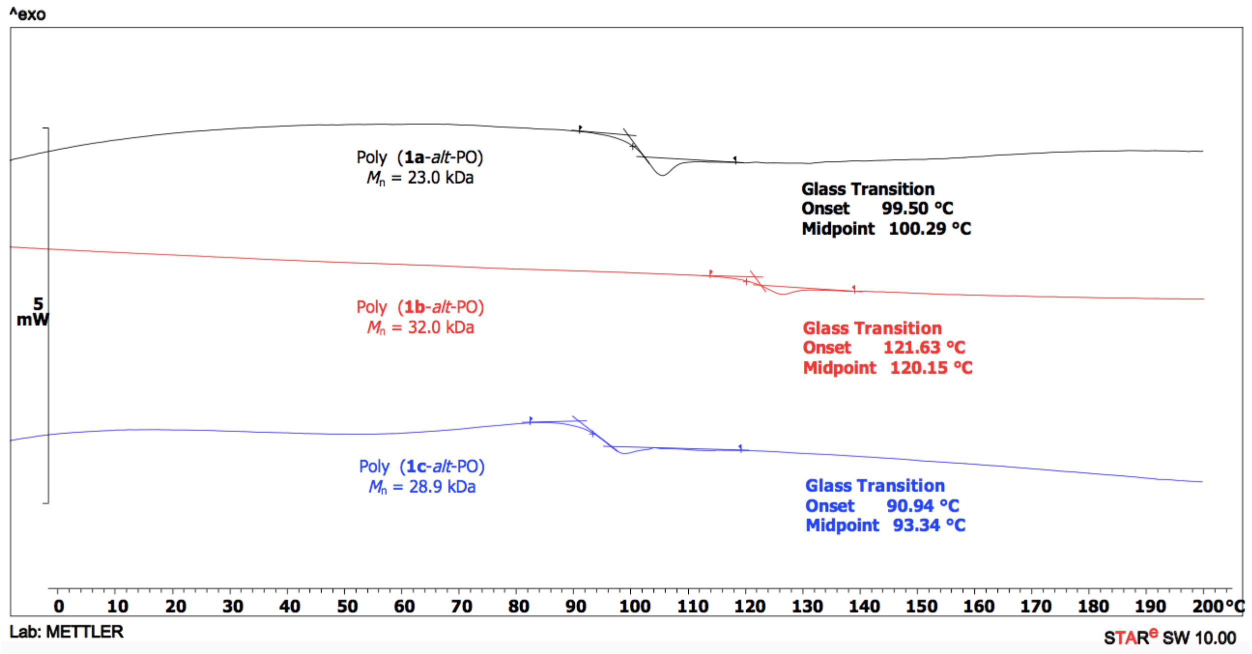

Supplementary Figure 25 | DSC thermograms of polymers in Supplementary Table 1, entry 4–6.

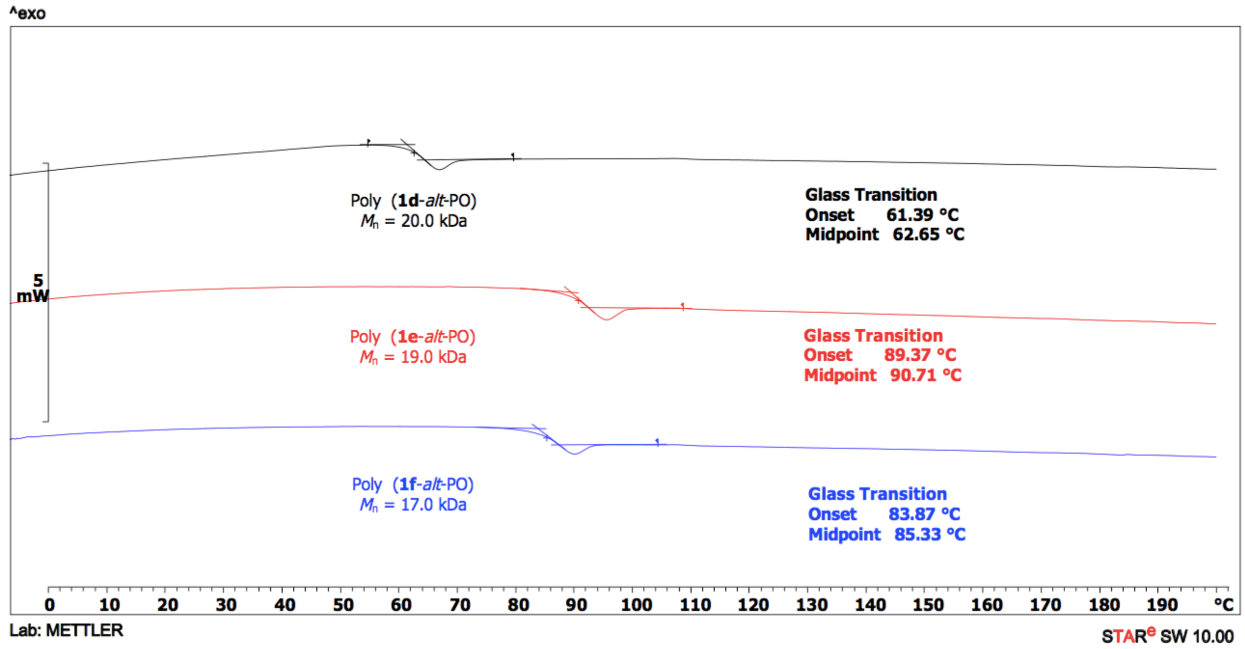

Supplementary Figure 26 | DSC thermograms of polymers in Supplementary Table 1, entry 7–9.

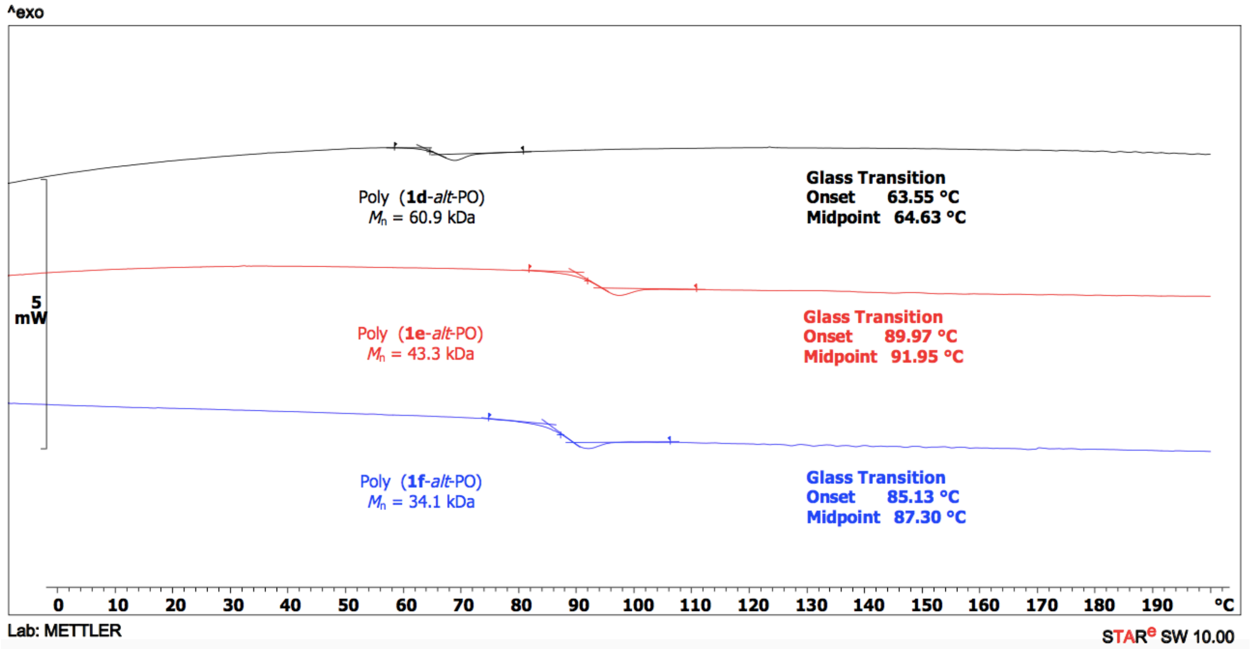

Supplementary Figure 27 | DSC thermograms of polymers in Supplementary Table 1, entry 10–12.

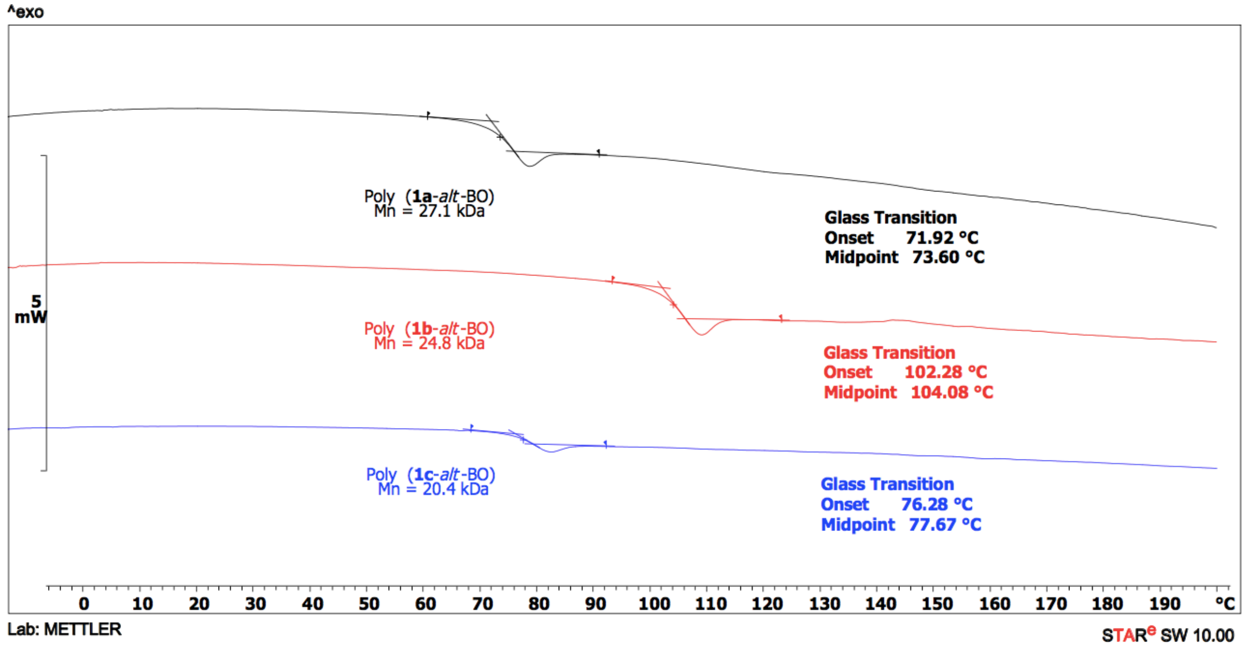

Supplementary Figure 28 | DSC thermograms of polymers in Supplementary Table 2, entry 1–3.

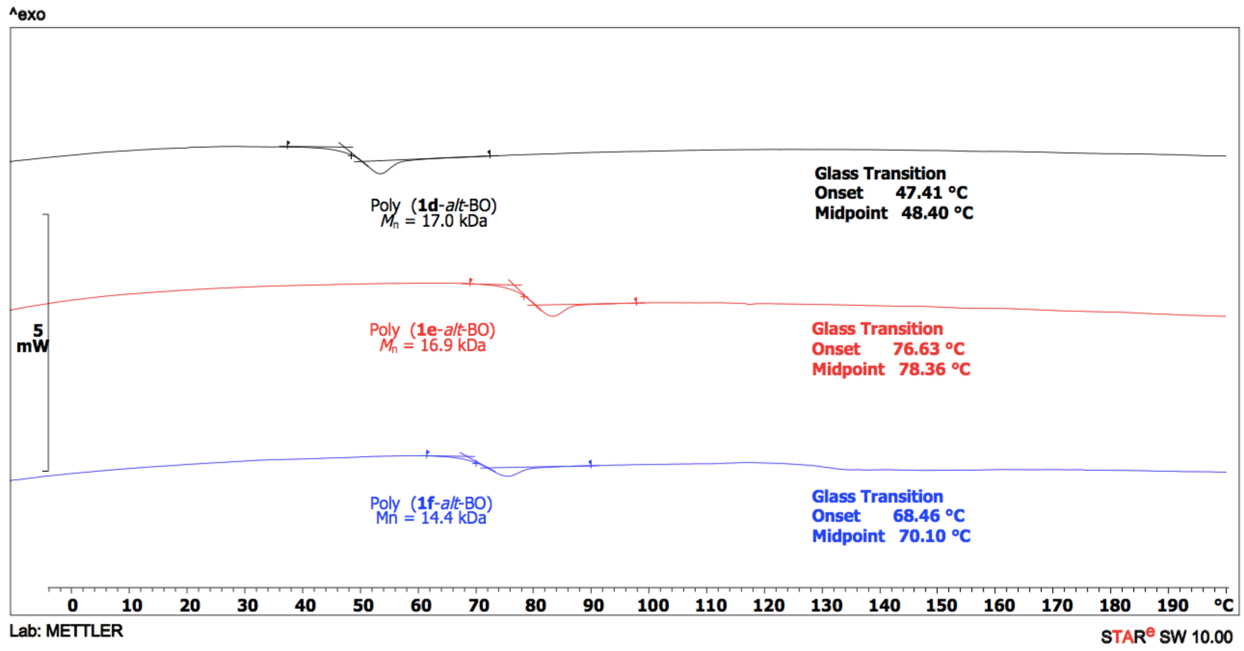

Supplementary Figure 29 | DSC thermograms of polymers in Supplementary Table 2, entry 4–6.

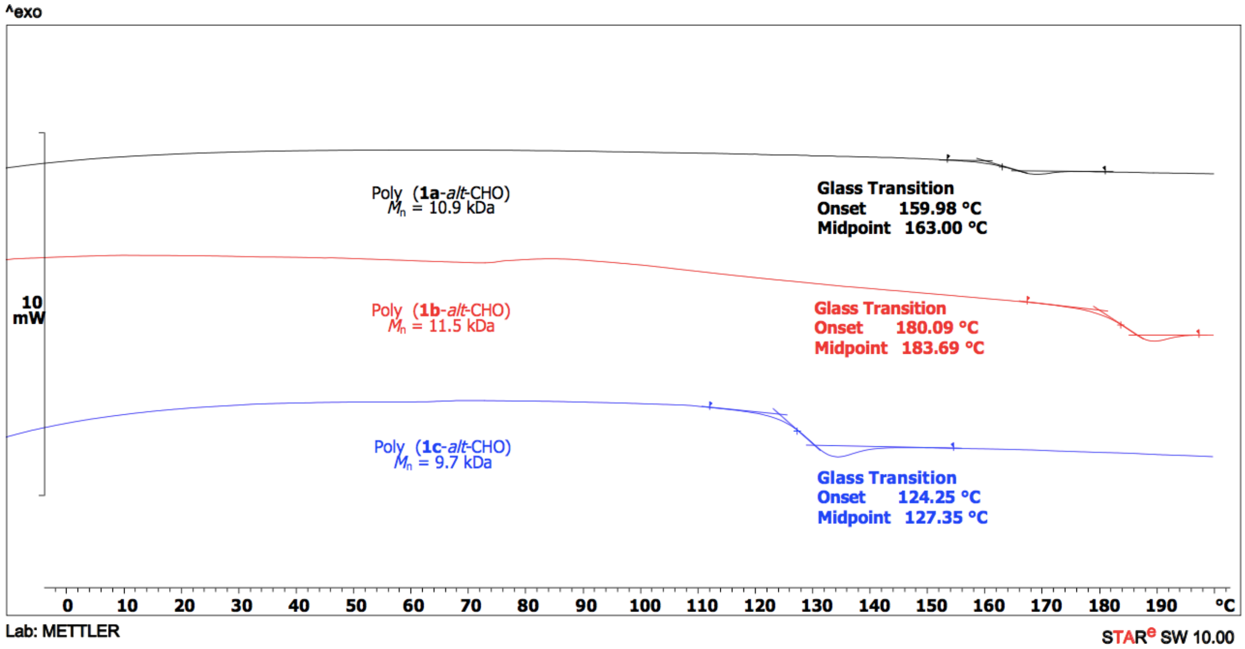

Supplementary Figure 30 | DSC thermograms of polymers in Supplementary Table 2, entry 7–9.

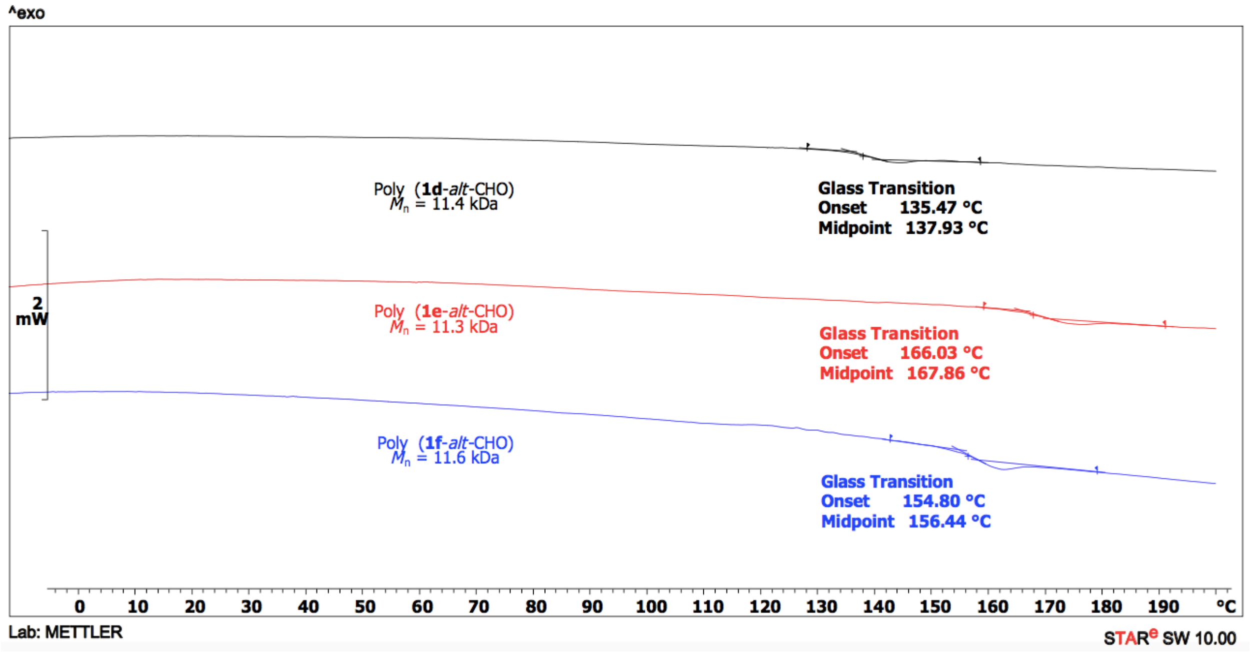

Supplementary Figure 31 | DSC thermograms of polymers in Supplementary Table 2, entry 10–12.

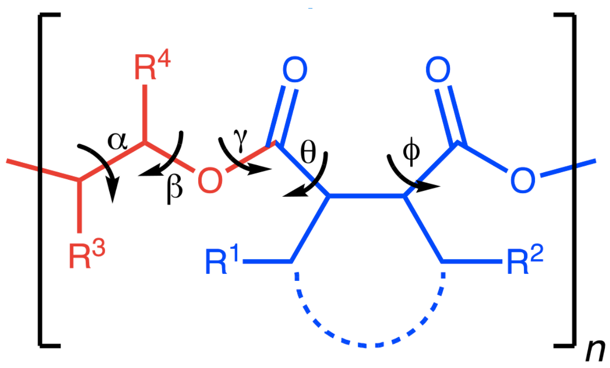

Supplementary Figure 32 | Schematic of the pertinent rotational degrees of freedom in the monomeric repeat units comprising the polyester systems considered in this work. Ramachandran-type plots depicting the full two-dimensional (2D) potential energy surfaces corresponding to dihedral rotations about θ and φ for each of the polyester model compounds (tricyclic: **2a** [$R^1 = R^2 = H$], **2b** [$R^1 = CH_3$, $R^2 = H$], **2c** [$R^1 = R^2 = CH_3$]; phthalic: **2d** [$R^1 = R^2 = H$], **2e** [$R^1 = CH_3$, $R^2 = H$], **2f** [$R^1 = R^2 = CH_3$]) are provided in Fig. 3c. The identity of R^3 and R^4 are determined by the choice of the epoxide comonomer as depicted in Fig. 2a.

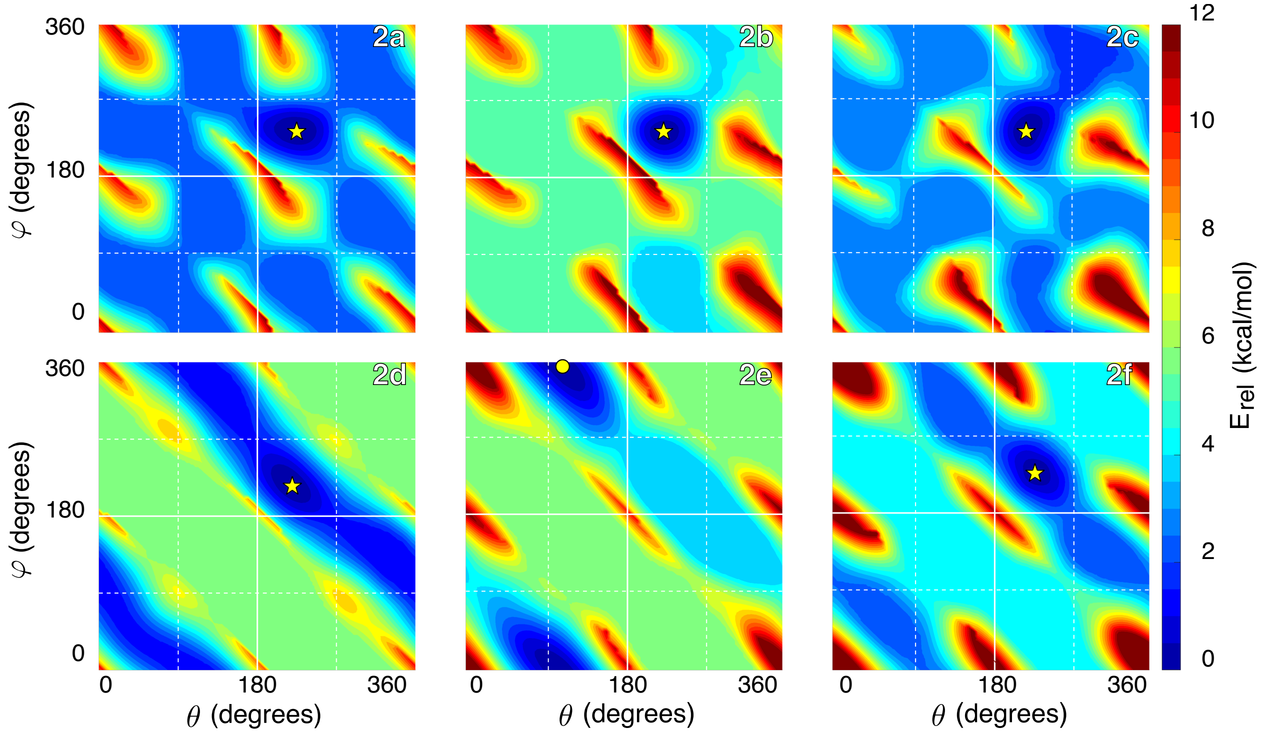

Supplementary Figure 33 | Area of the 2D rotational PES depicted in Fig. 3c that is accessible from the respective global minimum conformations of **2a-2f** as a function of the relative energy (E_{rel}) available for traversing rotational barriers.

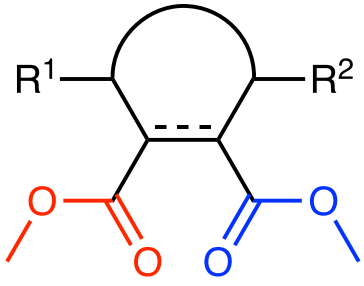

Supplementary Figure 34 | Graphical depiction of the model compounds **2a-2f** investigated in this work. For all intramolecular SAPT calculations, the left and right ester groups were defined by the atoms in red and blue, respectively. The interaction between the left (right) ester group and R^1 (R^2) is only computed when R^1 (R^2) is a methyl group.

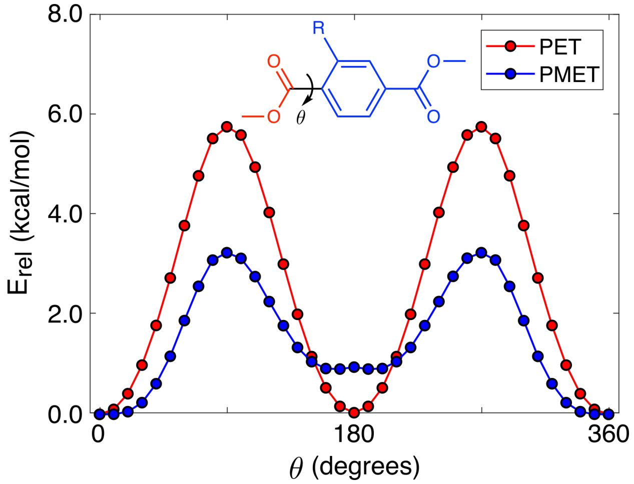

Supplementary Figure 35 | Potential energy diagram (computed at the B3LYP+D3(op)/6-311++G(d,p) level of theory) corresponding to dihedral rotations about θ in the PET (R = H) and PEMT (R = Me) model compounds depicted above.

Supplementary References

1. Van Zee, N. J., Sanford, M. J. & Coates, G. W. Electronic effects of aluminum complexes in the copolymerization of propylene oxide with tricyclic anhydrides: Access to well-defined, functionalizable aliphatic polyesters. *J. Am. Chem. Soc.* **138**, 2755–2761 (2016).
2. Sharma, S. et al. Synthesis and extraction studies with a rationally designed diamide ligand selective to actinide(IV) pertinent to the plutonium uranium redox extraction process. *Dalt. Trans.* **45**, 7737–7747 (2016).
3. Zhang, J., Lawrance, G. A., Chau, N., Robinson, P. J. & McCluskey, A. From Spanish fly to room-temperature ionic liquids (RTILs): Synthesis, thermal stability and inhibition of dynamin 1 GTPase by a novel class of RTILs. *New J. Chem.* **32**, 28–36 (2008).
4. Sanford, M. J., Peña Carrodeguas, L., Van Zee, N. J., Kleij, A. W. & Coates, G. W. Alternating copolymerization of propylene oxide and cyclohexene oxide with tricyclic anhydrides: Access to partially renewable aliphatic polyesters with high glass transition temperatures. *Macromolecules* **49**, 6394–6400 (2016).
5. Giri, R. & Yu, J.-Q. Synthesis of 1,2- and 1,3-dicarboxylic acids via Pd(II)-catalyzed carboxylation of aryl and vinyl C−H bonds. *J. Am. Chem. Soc.* **130**, 14082–14083 (2008).
6. Cao, D., Wang, C., Giesener, M. A., Liu, Z. & Stoddart, J. F. A rigid donor-acceptor daisy chain dimer. *Chem. Commun.* **48**, 6791–6793 (2012).
7. Tao, S. J. Positronium annihilation in molecular substances. *J. Chem. Phys.* **56**, 5499–5510 (1972).
8. Eldrup, M., Lightbody, D. & Sherwood, J. N. The temperature dependence of positron lifetimes in solid pivalic acid. *Chem. Phys.* **63**, 51–58 (1981).
9. Solomonik, E., Matthews, D., Hammond, J. R., Stanton, J. F. & Demmel, J. A massively parallel tensor contraction framework for coupled-cluster computations. *J. Parallel Distrib. Comput.* **74**, 3176–3190 (2014).
10. Malhotra, S. K. & Johnson, F. Steric interference in allylic and pseudo-allylic systems. II. Stereochemistry of exocyclic enolate anion protonation. *J. Am. Chem. Soc.* **87**, 5493–5495 (1965).
11. Johnson, F. Allylic strain in six-membered rings. *Chem. Rev.* **68**, 375–413 (1968).
12. Spiby, P., O'Neill, M. A., Duckett, R. A. & Ward, I. M. An infrared study of conformational-changes occurring during the drawing of PEMT, PET and PEMT/PET copolymer. *Polymer* **33**, 4479–4485 (1992).
